# Genome-wide association study identifies novel variants in olfactory, vitamin A, vitamin B, and cadherin pathways associated with learning and memory

**DOI:** 10.1038/s41598-025-32828-8

**Published:** 2025-12-18

**Authors:** Lloyd N. Hopkins, Nesli Avgan, Heidi G. Sutherland, Francesca E. Fernandez, Emma E. M. Knowles, Larisa M. Haupt, John Blangero, David C. Glahn, David H. K. Shum, Rod A. Lea, Lyn R. Griffiths

**Affiliations:** 1 Genomics Research Centre, Centre for Genomics and Personalised Health, School of Biomedical Sciences, Queensland University of Technology, Brisbane, QLD Australia; 2 School of Behavioural and Health Sciences, Faculty of Heath Sciences, Australian Catholic University, Banyo, QLD Australia; 3 Department of Psychiatry, Boston Children’s Hospital, Harvard Medical School, Boston, MA USA; 4 South Texas Diabetes and Obesity Institute, University of Texas Rio Grande Valley, Brownsville, TX USA; 5 Department of Rehabilitation Sciences and Research Institute of Smart Ageing, The Hong Kong Polytechnic University, Hong Kong, China

**Keywords:** Genetics, Neuroscience

## Abstract

Learning and memory, as fundamental components of human cognition, are heritable traits that are highly variable between individuals and within populations. Investigation into the genetic basis of cognition is a prominent area of research, with genetic associations being previously reported for a wide range of cognitive phenotypes. Here we utilise a genome-wide association study (GWAS) approach to evaluate the contribution of genetic variation to learning and memory phenotypes in a comprehensively phenotyped, well-characterised, healthy, and unrelated cohort of individuals (n = 613). Cognitive phenotypes were assessed using nine comprehensive test batteries consisting of twenty-one cognitive performance assessments including IQ, five measures for visual and verbal learning, and fifteen measures for semantic, working, episodic and prospective memory. Principal component analysis was utilised to amalgamate correlated test scores into additional new cognitive phenotypes. Our study identified genome wide significant associations for 13 loci across all phenotypes. A novel association was identified between the rs817826 SNP at 9q31.2 and verbal learning discrimination (p = 2.71 × 10^− 9^). GWAS of cognitive PCs identified three variants in the vicinity of thiamine (Vitamin B_1_) transporter gene *SLC19A3* (most significant SNP rs12105620, p = 2.17 × 10^− 9^), a 3’ UTR variant in *PPARD* (rs9658167, *p* = 1.47 × 10^− 8^), and an intronic variant in *RBFOX1* (rs17138790, *p* = 4.24 × 10^− 8^) associated with the cognitive PC related to visual and verbal learning. The cognitive PC relating to prospective and retrospective memory revealed a locus containing a synonymous variant in *NXPE3* (rs2305990, *p* = 6.56 × 10^− 9^) and intronic variants in *RD3* (rs17189035, *p* = 2.71 × 10^− 8^) and *WLS/GNG12-AS1* (rs17130484, *p* = 4.13 × 10^− 8^). Pathway analysis identified olfactory, vitamin A, and cadherin pathways as being significantly overrepresented across multiple cognitive domains. The novel associations identified provide candidates for further investigation and necessitate replication in similarly characterised independent cohorts.

**Supplementary Information:**

The online version contains supplementary material available at 10.1038/s41598-025-32828-8.

## Introduction

Memory is a cornerstone of cognitive function, acting as a sophisticated process that integrates sensory inputs and experiences to facilitate adaptive responses to information and stimuli. The fundamentals of memory are shaped by an intricate interplay of genetic and molecular factors, which over time have become more implicit as more is revealed about the foundation of human cognition. Cognition can be stratified into a number of domains – Sachdev et al. (2014) identify learning and memory, language, perceptual function, executive function, attention, and social cognition as six major neurocognitive domains^1^. Developing the understanding of these domains within a molecular genetic context may provide translatable insights into the treatment of disease-induced impairment, as well as personalised health insights for otherwise healthy individuals.

Genetic variability has been long understood to play a pivotal role in cognitive function, with large-scale studies using publicly available genetic datasets being undertaken. Cognition is a complex trait and is highly heritable, with studies indicating substantial genetic influences accounting for more than 80% of variability^2^. As genetic factors become more evident and datasets more comprehensive, genome-wide association studies (GWAS) have continued to implicate novel loci to explain genetic variability in cognitive differences^3–5^. A recent GWAS meta-analysis by Savage et al. comprising of 269,867 individuals has most recently identified 205 distinct genomic loci associated with a multidimensional set of cognitive performance tests^6^. Another large study of 300,486 individuals identified 709 genes and 148 independent loci associated with general cognitive function^7^. GWAS with larger sample sizes are required to continue to identify novel loci for investigation; however, smaller cohort studies with accurately reported phenotypes and greater uniformity in sample collection and processing methods can provide useful insight into the polygenic nature of cognition^8–10^.

Previous studies have evaluated the association of specific measures and batteries of tests with genetic variability, while neglecting to consider the relationships between tests and phenotypes. Principal component analysis (PCA) can be utilised to constellate sets of correlated variables into a set of new uncorrelated variables – principal components (PCs). These PCs retain the fundamental patterns of the grouped variables while reducing the dimensionality of the data, discarding the less relevant features, and revealing trends that may not be initially perceived^11–13^. This process has been utilised in numerous GWAS to highlight features that would have otherwise not been discernible^5,7,14^, and can provide significant insight into the identification of genetic variability within a cohort.

In this study, we investigated associations between genetic variants and cognitive measures in a healthy cohort, quantified using measures of intelligence, learning, and memory using a representative battery of standardised and validated tests. To uncover novel evidence implicating genetic variability in cognitive function, GWAS were performed for each individual test of a specific memory type, as well as for the principal components of cognitive measures. Genes and pathways related to known molecular processes and brain functions were implicated in this study, providing insights into the nature of cognition and informing future research. The deep phenotyping of cognition, using multiple standardised tests, and subsequent PCA of these measures supports PCA as a valid data reduction method in GWAS for correlated cognitive phenotypes.

## Methods

### Study population and cognitive phenotypes

The study was approved by both the Griffith University (MSC/01/09/HREC) and Queensland University of Technology (1300000486) Human Research and Ethics Committees and all experiments were performed in accordance with relevant guidelines and regulations. A cohort of 619 healthy participants were recruited from the Southeast Queensland region of Australia, principally the Brisbane and Gold Coast areas, through public advertisement. There were no age or education criteria, and participants with a history of psychiatric disorder or head injury were excluded to preserve the cohort’s capacity as a representative sample of cognitive and memory without additional confounding factors. All subjects provided written informed consent prior to collection and testing. Saliva samples were collected from all participants immediately post completion of all memory testing for genetic analysis using Oragene DNA Self Collection kits (DNA Genotek Inc., Ottawa, ON, Canada) with DNA extraction performed as per manufacturers protocol.

Memory, learning, and intelligence phenotypes were evaluated using eight cognitive battery tests as presented in Table 1 and as previously described^15–17^. All participants were assessed in a quiet, well-lit room by the same examiner, with a total of 21 measures recorded for all participants (Table 1). Principal component analysis (PCA) was applied to these 21 measures to transform the data into cognitive PCs using R (4.3.1) packages factoextra^18^ and FactoMineR^19^, with the first 3 PCs selected for further analysis after identifying the inflection point of the scree plot of the eigenvalues.

**Table 1:** Cognitive domains and associated cognitive measures assessed in this study.

Intelligence
*IQ*
Wechsler Abbreviated Scale of Intelligence (WASI) (WASI IQ)^20^.
**Learning**
*Visual Learning*
Shum Visual Learning Test (SVLT) Learning Index (SVLT LI), Retention after Interference (SVLT RII), and Delayed Retention (SVLT DRI)^21,22^.
*Verbal Learning*
Hopkins Verbal Learning Test (HVLT) Learning (HOP LEARN), Retention (HOP RETEN), and Discrimination (HOP DISCRIM)^23^.
**Memory**
*Semantic Memory*
Wechsler Adult Intelligence Scale (WAIS) III – Information (WAIS INFO)^24^.
*Working Memory*
WAIS III – Letter Number Sequencing (WAIS LNST)^24^.
*Verbal Memory*
HVLT Recall (HOP RECALL), and Delay (HOP DELAY)^23^.
*Visual Memory*
Wechsler Memory Scale III - Visual Reproduction (VISREP) Test 1 (Immediate) (VISREP IMD) and Test 2 (Delayed) (VISREP DELAY)^25^. SVLT Overall Learning Score (SVLT OLS)^21,22^.
*Retrospective Memory*
Prospective and Retrospective Memory Questionnaire (PRMQ) Retrospective Memory (PRMQ RM)^26^.
*Prospective Memory*
PRMQ Prospective Memory (PRMQ PM)^26^. Comprehensive Assessment of Prospective Memory (CAPM) Instrumental Activities of Daily Living (CAPM IADL), Basic Activities of Daily Living (CAPM BADL), and sum of subscales (CAPM TOTAL)^27,28^. Memory for Intentions Screening Test (MIST) Immediate (MIST IMD) and Delay (MIST DELAY)^29^.

### Genotyping and quality control

Background information was reviewed by the study team for participants to confirm that all met inclusion criteria as listed above, with six individuals being removed from the study for not satisfying the criteria. The remaining 613 samples were genotyped on Illumina Human OmniExpress-24 BeadChip Arrays (v1.0 and v1.1) (Illumina, Inc., San Diego, CA, USA). Only the overlapping SNPs in both arrays were included in the study (709,354 SNPs) to maintain a high genotyping rate. Four samples were excluded post genotyping due to the overall genotyping call rate being less than 95%.

Samples and SNPs were quality controlled and filtered using PLINK (v1.90b7)^30^, based on minor allele frequency > 0.01, Hardy-Weinberg equilibrium p-value > 0.001, genotyping call-rate > 0.95, and failure rate per-individual < 0.05. After quality control procedures, 598 individuals and 624,763 SNPs were retained. One individual recorded a missing WASI IQ measure – population characteristics were analysed with the full remaining cohort of 598 individuals, with the GWAS being performed with this individual excluded for a total of 597 individuals.

After quality control, population structure characteristics were analysed using PLINK (v1.90b7) and R packages factoextra, FactoMineR, and visualised using ggplot2^31^. Population characterisation, performed using PCA, was used to elucidate the genetic substructure of the population and compared to self-reported ethnicity, the latter being utilised as a covariate in the association analysis. Correction for population substructure is essential in GWAS analysis to control for the structure of the cohort, and although self-reported ethnicity can assist in the identification and stratification of the population structure, it can be optimal to utilise the PCs of the allele frequencies as covariates to correct for heterogeneity in lieu of ethnicity^11^. The first 3 PCs were included as covariates after identifying the inflection point of the eigenvalues for the PCs of genetic substructure using the scree plot. Imputation was not considered for this dataset as the study cohort comprised of a range of ethnicities that are not included in the most widely used reference panels, reducing the probability of correct haplotype matches.

### Genome-wide association analyses

Genomic inflation and association analyses were performed using PLINK (v1.90b7). An additive linear model to test association was fitted to each individual phenotype and to the PCs of the cognitive measures, with sex, age, IQ, and the first 3 PCs of the genetic substructure applied as covariates. For the WASI IQ and cognitive PC association analyses IQ was not included as a covariate as to not confound the analysis of these phenotypes. To account for multiple testing the genome wide significance threshold was set at the widely accepted *p* < 5 × 10^− 8^, with a suggestive threshold set at *p* < 1 × 10^− 5^.

### Replication cohorts

To validate the findings, results were compared with two replication cohorts: one consisting of a subset of the QIMR Twin cohort^32^ and the second being the Genetics of Brain Structure and Function (GOBS) study cohort^33,34^. In the QIMR Twin cohort, intelligence, semantic memory, and working memory scores was assessed in a cohort of Australian dizygotic and monozygotic twins of European ancestry using the same tests with the current study cohort; WASI IQ, WAIS INFO, and WAIS LNST respectively. Intelligence and semantic memory results were obtained from 2598 individuals and working memory results from for 956 individuals. Genotyping was performed using a combination of Illumina 317 K, Illumina 370 K, and Illumina 610 K microarray chips and GWAS was performed using METAL for 7,681,669 markers. In the GOBS study, 1709 participants of Mexican and American ancestry were genotyped using Illumina Human1Mv1, Human1M-Duov3, HumanHap550v3, and HumanExon510Sv1 BeadChips as previously described^35^. Each participant completing a battery of tests consisting of standardised measures^36^, with phenotypes equivalent to those assessed in this study compared for suggestively significant variants. Only data related to the associations with the cognitive measures, not the cognitive PC’s, was available for this cohort. A significance level of *P* < 0.05 was used for the replication analysis given the small sample size of the replication cohorts.

### Pathway analysis

Pathway analysis was performed on the linear association outputs by collating all 624,763 variants into SNP-sets using PLINK. Loci were mapped to corresponding genes by genomic location using the hg19 reference genome. A 50 kb window around each gene was defined, an approach previously described to include SNPs within intergenic regulatory regions neighbouring genes, without sacrificing substantial specificity^37–39^. This set-based method was selected in lieu of tools that use a reference genome and calculate linkage disequilibrium (LD) against a reference dataset as the study population is un-imputed and of mixed ethnicity. Set-based methods allow for the consideration of multiple SNPs within a gene that alone may not be highly significant but could have greater combined genetic effects^40^. The set-based p-values for each gene were calculated using PLINK adaptive permutation, pruning SNPs in high LD, and the outputs subsequently analysed using the R package ActivePathways^41^ to produce overrepresented pathways. Gene Ontology (GO)^42^, Panther^43^, and Reactome^44^ databases were queried, and pathways were filtered to those within the range of 10 to 200 genes to minimise bias related to size and increase specificity in the pathway analysis; this range being well documented in previous studies^39,45^.

## Results

### Study population characteristics

Summary statistics were generated for the 598 participants with cohort demographics presented in Table 2. The majority of participants were female (71.4%) with a median age of 20 (Mean age 23, SD 8.0) and an age range of 16–65. One individual recording a missing WASI_IQ measure was excluded from downstream analysis. Genomic inflation was calculated as 1.02 overall for the 21 measures and 1.00 for the 3 cognitive PCs in the multivariate models (QQ plots provided in Supplementary Fig. 1). The population substructure (Supplementary Fig. 2) demonstrated that self-reported ethnicity did not distinctly cluster and indicated unreported mixed ethnicity for some individuals, supporting the use of genetic PCs in the stratification of the population structure.

**Table 2:** Summary statistics of cohort.

Variable		Participants (*n* = 598)	
Age			
	Median (IQR)	20	(18,24)
	Range	16–65	
	Mean (SD)	23	(8.03)
		N	(%)
Age Group			
	16–25	466	(77.9%)
	26–35	79	(13.2%)
	36–45	36	(6.0%)
	46–55	10	(1.7%)
	> 55	7	(1.2%)
Sex			
	Male	171	(28.6%)
	Female	427	(71.4%)
Self-Reported Ethnicity			
	Caucasian/Australian	448	(74.9%)
	European	46	(7.7%)
	Mixed	32	(5.4%)
	Asian	23	(3.8%)
	Other	49	(8.2%)

### Principal component analysis of cognitive phenotypes

**Fig. 1:**
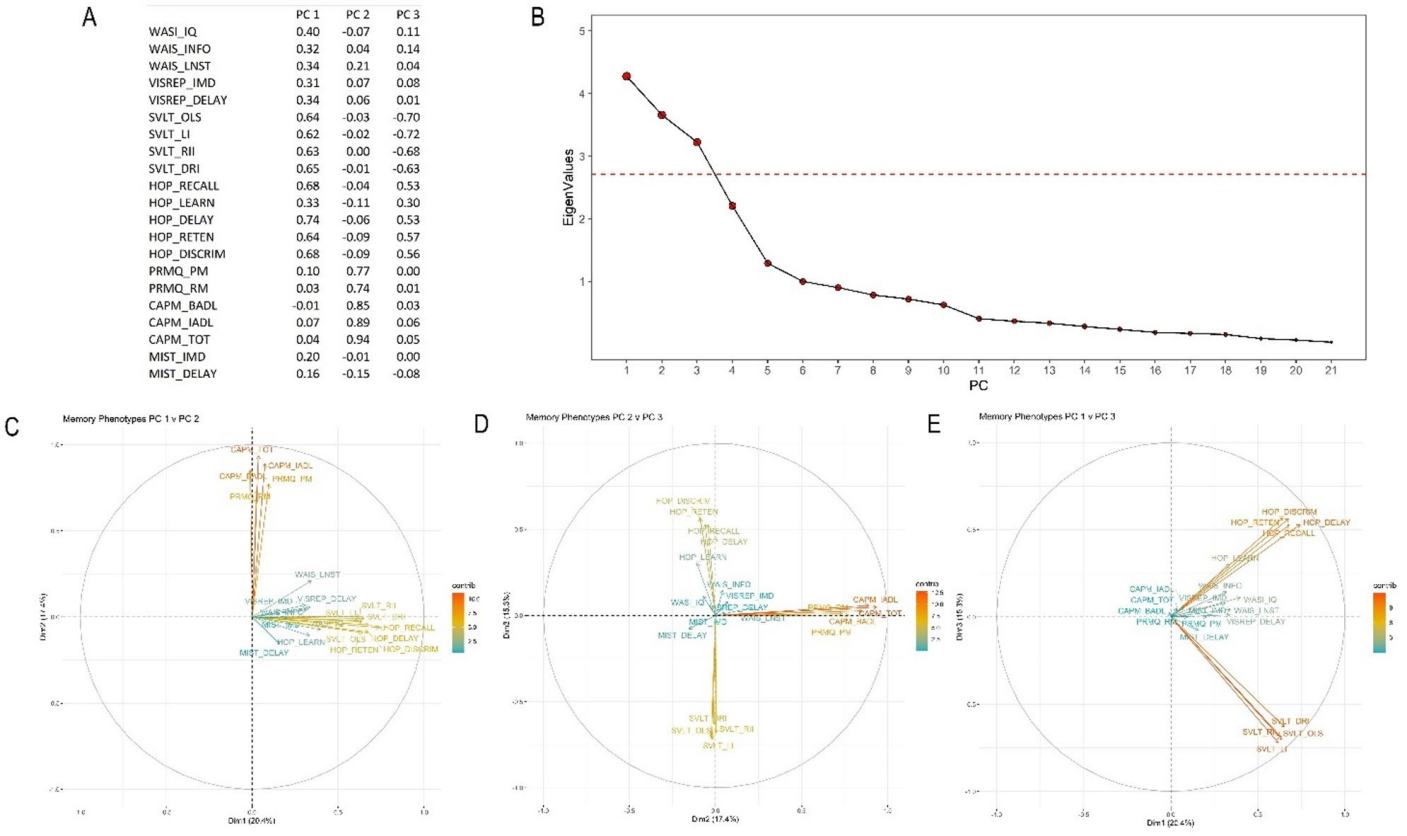
PCA of cognitive measures. **A**: Summary of the first 3 PCs for the 21 cognitive measures. **B**: Scree plot of eigenvalues depicting variance of explained by PCs of cognitive measures, inflection point denoted by red dashed line. C: Loading plot of PC1 and PC2 of 21 cognitive measures to identify which measures have the largest effects of each component. D: Loading plot of PC2 and PC3. E: Loading plot of PC1 and PC3.

Twenty-one measures were transformed using PCA, with underlying variation illustrated in Fig. 1. The first 3 cognitive PCs explained 52% of the phenotype variance (Figs. 1 A and B). Cognitive PC1 explained 20% of the variance, with positive loading evident for HVLT and SVLT test batteries (0.33 to 0.74), representing visual and verbal learning (Figs. 1 A and C). Cognitive PC2 explained 17% of variance, with high positive loadings (0.74 to 0.94) observed for CAPM and PRMQ test batteries, representing prospective and retrospective memory (Figs. 1A and D), while PC3 explained 15%, with negative loading for SVLT (−0.63 to −0.72) and positive loading for HVLT (0.30 to 0.57) test scores (Figs. 1A and E).

### Genome-wide association results

**Fig. 2:**
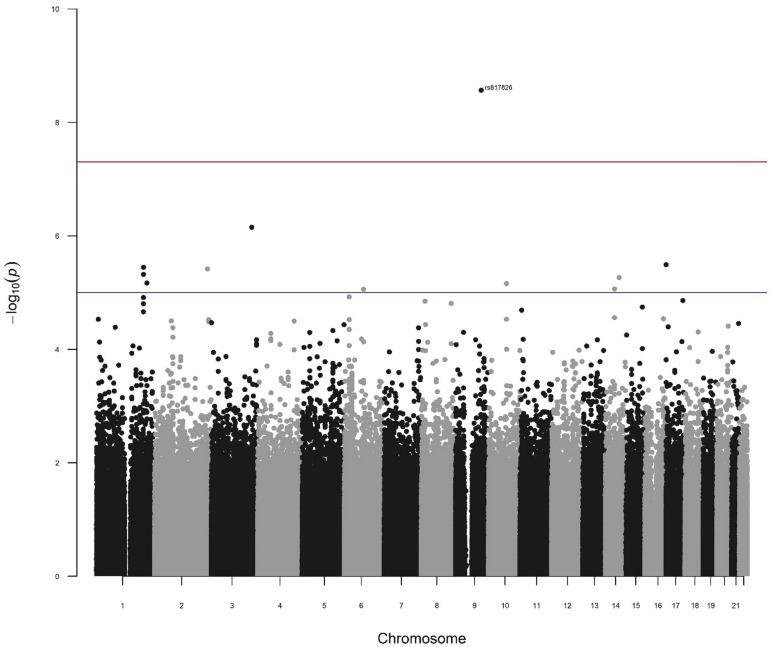
Manhattan plot for the HOP DISCRIM measure, with significant SNP rs817826 marked (*p* = 2.71 × 10^− 9^). The genome wide significance threshold (*p* < 5 × 10^− 8^) is indicated by the red line. The blue line indicates the suggestive threshold (*p* < 1 × 10^− 5^).

**Fig. 3:**
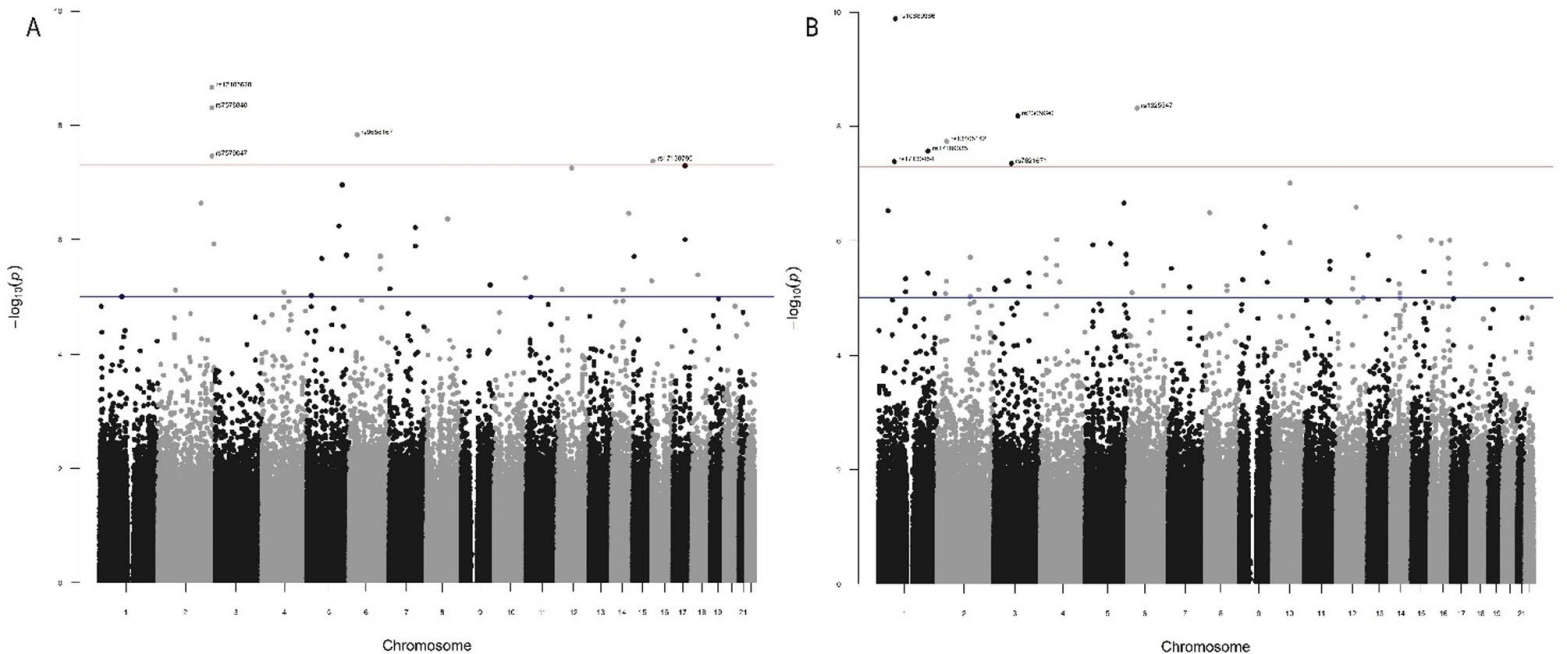
Manhattan plots of the GWAS results of the PCA of the cognitive measures, with loci reaching the genome wide significance threshold (*p* < 5 × 10^− 8^) indicated by the red line. The blue line indicates the suggestive threshold (*p* < 1 × 10^− 5^). Significant SNPS are denoted in Table 3. **A**: Plot of PC1 extracted from the cognitive measures. **B**: Plot of PC2 extracted from the cognitive measures.

Linear regression analysis was completed for 624,763 autosomal SNPs in a cohort of 597 healthy individuals adjusted for sex, age, IQ, and the first 3 PCs of the genetic substructure. For the WASI IQ and cognitive PC association analyses IQ was not included as a covariate. The multi-covariate additive model was fitted for each memory phenotype and the PCs of the phenotypes. In total, 13 SNPs passed the genome wide significance threshold of *p* < 5 × 10^− 8^ (Table 3), and 289 SNPs passed the suggestive threshold (*p* < 1 × 10^− 5^) across all 24 phenotypes (Supplementary Table 1). Manhattan plots of the association results for all phenotypes that show genome wide significant SNPs are presented in Figs. 2 and 3. One SNP reached the genome-wide significance threshold for the cognitive measures, rs817826, significantly associated with the HOP_DISCRIM measure for verbal learning (*p* = 2.71 × 10^− 9^), with the remaining 12 SNPs being significant for cognitive PC1 and cognitive PC2. No SNPs were significant at *p* < 5 × 10^− 8^ for PC3. Manhattan plots for the association results with no genome-wide significant SNPs are presented in Supplementary Figs. 3 and 4.

**Table 3:** Genome wide significant SNPs (*p* < 5 × 10^− 8^) identified in the GWAS. MAF is for GRC GOM cohort. GRCh37 hg19 genome Assembly.

Phenotype	SNP	Chrom.	Position (BP, GRCh37)	Effect Allele (A1)	MAF (A1)	Gene	*N*	BETA	*P*-value
PC 2	rs17130484	1	68,581,840	C	0.060	*WLS/GNG12-AS1* (intron)	592	−1.22	4.13 × 10^− 08^
PC 2	rs10889966	1	73,064,758	G	0.016	(upstream *NEGR1*)	593	−2.69	1.30 × 10^− 10^
PC 2	rs17189035	1	211,657,371	A	0.034	*RD3* (intron)	595	−1.57	2.71 × 10^− 08^
PC 2	rs13405142	2	41,556,281	A	0.024	(intergenic *SLC8A1*-*PKDCC*)	597	−1.91	1.83 × 10^− 08^
PC 1	rs7579047	2	228,535,921	A	0.016	(downstream *SLC19A3*)	597	−2.94	3.44 × 10^− 08^
PC 1	rs7578840	2	228,546,339	A	0.014	(downstream *SLC19A3*)	596	−3.20	4.95 × 10^− 09^
PC 1	rs12105620	2	228,548,897	G	0.012	*SLC19A3* (3’UTR)	597	−3.32	2.17 × 10^− 09^
PC 2	rs7621671	3	74,906,533	G	0.011	(upstream *CNTN3*)	594	−2.97	4.44 × 10^− 08^
PC 2	rs2305990	3	101,520,537	A	0.010	*NXPE3* (synonymous)	596	−3.25	6.56 × 10^− 09^
PC 1	rs9658167	6	35,394,080	A	0.013	*PPARD* (3’UTR)	597	−2.99	1.47 × 10^− 08^
PC 2	rs1325547	6	40,232,556	A	0.011	(downstream *LRFN2*)	597	−3.16	4.79 × 10^− 09^
HOP_DISCRIM	rs817826	9	110,156,300	G	0.156	(intergenic *RAD23B*-*KLF4*)	593	−0.37	2.71 × 10^− 09^
PC 1	rs17138790	16	5,928,608	C	0.029	*RBFOX1* (intron)	597	−1.94	4.24 × 10^− 08^

### Replication analysis of genome-wide association results in two independent cohorts

Replication analysis of genome-wide and suggestively significant (*p* < 1 × 10^− 5^) associations was undertaken in two independent cohorts with similar cognitive measures: the QIMR Twin cohort consisting of Australian twins of European ancestry, and the GOBS cohort consisting of Mexican-American pedigrees. Summary statistics were assessed for same or similar measures where available. Correlation between suggestively significant (*p* < 1 × 10^− 5^) SNPs in the GRC GOM cohort and nominal significant SNPSs (*p* < 0.05) the QIMR Twin cohort identified one locus, rs9948718, located downstream from *MAPK4*, as being associated with working memory in the QIMR Twin cohort and GRC GOM cohort (Table 4). Nominal associations were also identified between cognitive PCs and WASI_IQ, WAIS_LNST, and WAIS_INFO results. In the GOBS cohort, proximate SNPs rs1032704 and rs4931672 at 12p11.21 were nominally significant for the Penn Conditional Exclusion Test (PCET) Face Memory Test and reached the suggestively significant threshold in the GRC GOM cohort for the SVLT LI measure for visual learning (Table 5). Variant rs6035340, an intronic variant in *SLC24A3*, was nominally significant for the California Verbal Learning Test (CVLT) Delay Test and reached the suggestively significant threshold in the GRC GOM cohort for prospective memory measured by the MIST DELAY test. Replication data for the genome-wide significant SNPs is included in Supplementary Table 2. Of note, two SNPs, rs17189035 and rs17130484, located within the introns of the *RD3* and *WLS/GNG12-AS1* genes respectively, reached the genome wide significance threshold (*p* < 5 × 10^− 8^) in the GRC GOM cohort for cognitive PC2 and *p* < 0.05 in the QIMR Twin cohort.

**Table 4:** Suggestively significant (*p* < 1 × 10^− 5^) SNPs from the GRC GOM cohort that reach nominal significance (*p* < 0.05) in the QIMR twin cohort and their associated cognitive measures.

Phenotype	Chrom.	SNP	GOM Effect Size (β)	GOM *P*-value	GOM MAF	Replication Test	Replication Effect Size (β)	Replication *P*-value	Replication MAF
Memory Based Cognitive Tests									
WAIS_LNST	18	rs9948718	0.89	8.25 × 10^− 6^	0.146	WAIS_LNST	0.40	3.96 × 10^− 2^	0.132
PCs									
PC 2	1	rs17130484	−1.22	4.13 × 10^− 8^	0.060	WASI_IQ	2.03	8.64 × 10^− 3^	0.065
PC 2	1	rs17189035	−1.57	2.71 × 10^− 8^	0.034	WAIS_LNST	−0.59	3.52 × 10^− 2^	0.044
PC 2	3	rs1516398	−0.59	7.14 × 10^− 6^	0.232	WASI_IQ	−0.95	3.39 × 10^− 2^	0.244
PC 2	3	rs2728030	−0.59	6.80 × 10^− 6^	0.231	WASI_IQ	−0.92	3.81 × 10^− 2^	0.245
PC 3	4	rs1438371	−1.86	4.36 × 10^− 7^	0.021	WASI_IQ	3.32	4.52 × 10^− 2^	0.017
PC 1	4	rs1438371	−1.90	8.36 × 10^− 6^	0.021	WASI_IQ	3.32	4.52 × 10^− 2^	0.017
PC 1	5	rs17830827	−1.22	2.15 × 10^− 6^	0.055	WASI_IQ	−2.39	3.66 × 10^− 3^	0.064
PC 1	5	rs2071161	−0.98	1.87 × 10^− 6^	0.089	WAIS_LNST	0.41	2.55 × 10^− 2^	0.097
PC 1	5	rs11739136	−0.98	1.93 × 10^− 6^	0.090	WAIS_LNST	0.40	2.89 × 10^− 2^	0.098
PC 2	8	rs10808637	−1.35	6.06 × 10^− 6^	0.031	WAIS_LNST	−0.64	1.25 × 10^− 2^	0.035
PC 2	8	rs10092485	−1.31	7.49 × 10^− 6^	0.033	WAIS_LNST	−0.60	1.96 × 10^− 2^	0.035
PC 1	9	rs16910952	−1.89	6.24 × 10^− 6^	0.021	WAIS_INFO	−0.99	4.82 × 10^− 3^	0.026
PC 2	9	rs7029019	−1.29	4.80 × 10^− 6^	0.044	WAIS_LNST	−0.67	1.76 × 10^− 2^	0.035
PC 2	9	rs16930907	−0.96	5.29 × 10^− 6^	0.072	WAIS_INFO	0.67	2.73 × 10^− 2^	0.044
PC 2	12	rs7301862	−1.12	2.60 × 10^− 7^	0.062	WASI_IQ	−1.54	3.66 × 10^− 2^	0.071
PC 2	14	rs9323346	−0.61	8.20 × 10^− 6^	0.195	WASI_IQ	−1.88	1.48 × 10^− 4^	0.192
PC 3	14	rs12888097	−0.54	5.53 × 10^− 6^	0.252	WAIS_INFO	0.29	4.63 × 10^− 2^	0.223
PC 1	17	rs12936983	−1.42	1.00 × 10^− 6^	0.045	WASI_IQ	−1.86	4.77 × 10^− 2^	0.042

Bolded lines show SNPs that are below the nominal significance threshold in the QIMR Twin cohort with the same directional effect as the GRC GOM cohort.

**Table 5:** Suggestively significant (*p* < 1 × 10^− 5^) SNPs from the GRC GOM cohort that reach nominal significance (*p* < 0.05) in the GOBS cohort and their associated cognitive measures.

Phenotype	Chrom.	SNP	GOM Effect Size (β)	GOM *P*-value	GOM MAF	Replication Test	Replication Effect Size (β)	Replication *P*-value	Replication MAF
SVLT_LI	12	rs1032704	0.11	3.2 × 10^− 6^	0.452	PCET Face Memory Test	0.08	3.22 × 10^− 2^	0.456
SVLT_LI	12	rs4931672	0.11	2.5 × 10^− 6^	0.452	PCET Face Memory Test	0.08	2.74 × 10^− 2^	0.458
MIST_DELAY	20	rs6035340	2.33	7.93 × 10^− 6^	0.458	CVLT Delay	0.07	3.68 × 10^− 2^	0.460

### Gene-set based pathways analysis

**Fig. 4:**
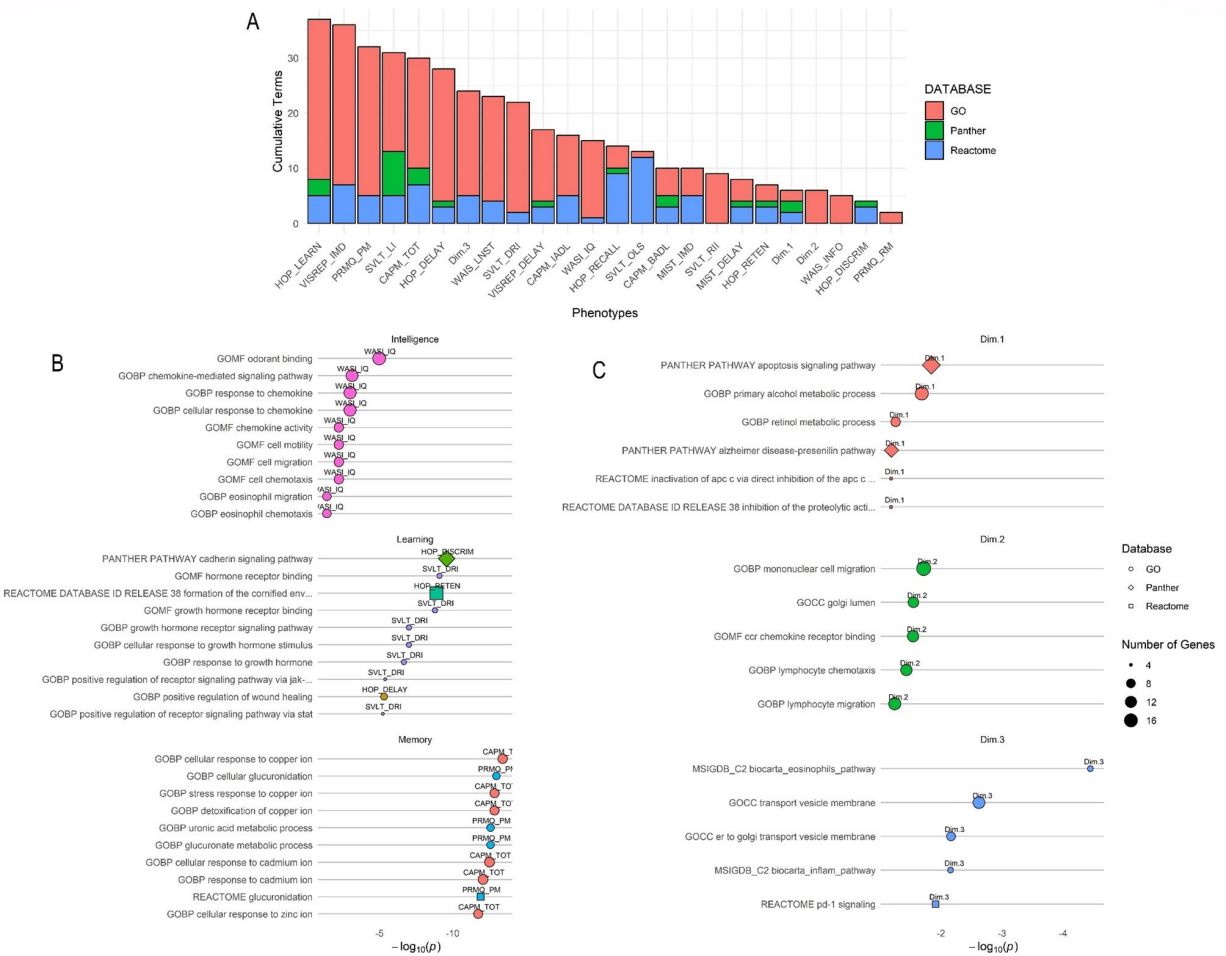
Results for Gene Ontology (GO), Panther, and Reactome pathway analysis across all phenotypes. All reported p-values are adjusted by Holm–Bonferroni correction. **A**: Cumulative count of significant (*p* < 0.1) terms for all phenotypes. **B**: Top significant terms for each cognitive domain C: Top significant terms for each cognitive PC extracted from the cognitive measures.

To assess if individual loci were related to specific genes and pathways, gene-set pathways analysis was performed. Loci were mapped to genes, assessed for over-representation against cognitive phenotypes, and the resulting gene-sets analysed for enrichment against pathways. The pathway analysis results identified the most significantly associated pathways (*p* < 0.1) for the gene-sets for each phenotype. Figure 4 A summarises the cumulative number of significant pathways for each phenotype, with Figs. 4B and C illustrating the most significant pathways for each cognitive domain and for cognitive PCs 1–3. An average of 18 significant (Holm–Bonferroni corrected *p* < 0.1) pathways from the three databases were identified for each cognitive test (range: 2 to 37), and an average of 12 per cognitive PC (range: 6 to 24). The most significant pathways were identified to be related to cellular responses to ions, namely copper (GO:0071280, *p* = 3.93 × 10^− 14^), cadmium (GO:0071276, *p* = 3.07 × 10^− 13^), and zinc (GO:0071294, *p* = 1.80 × 10^− 12^) in the CAPM TOT test phenotype representing prospective memory. As for other test batteries, cadherin signalling (PANTHER PATHWAY: P00012, *p* = 2.63 × 10^− 10^) and sensory perception of taste (GO:0050913, *p* = 2.90 × 10^− 5^), and odorant binding (GO:0005549, *p* = 1.59 × 10^− 3^) pathways were identified to be overrepresented in HVLT outcomes, glucuronidation (GO:0052695, *p* = 1.02 × 10^− 13^), uronic acid (GO:0006063, *p* = 2.59 × 10^− 13^), retinoic acid (GO:0001972, *p* = 1.74 × 10^− 10^), and cadherin signalling (PANTHER PATHWAY: P00012, *p* = 7.17 × 10^− 9^) pathways identified in PRMQ test phenotypes, and growth hormone receptor binding (GO:0005131, *p* = 1.62 × 10^− 9^) identified in SVLT outcomes. Additional pathway results are presented in Supplementary Fig. 5 and Supplementary Table 3.

## Discussion

The evolving landscape of genetic factors in cognitive research is marked by novel findings in both large-scale population-based studies and in smaller cohort studies, both providing insight into the complex phenotypes of memory and cognition. In this current study, we have performed a GWAS in a cohort of healthy individuals, who were extensively phenotyped for a range of cognitive traits, to discover genetic variants involved in intelligence, learning, and memory. As well as individual test scores, PCA was also utilised to extract underlying latent patterns in the data, allowing for more comprehensive, muti-phenotype analysis of the dataset. Results revealed that 13 SNPs passed the genome wide significance threshold of *p* < 5 × 10^− 8^ and 289 SNPs passed the suggestive threshold of *p* < 1 × 10^− 5^ across all 24 phenotypes.

A genome-wide significant association was identified between HVLT recall score, measuring verbal memory, and variant rs817826 in the GRC GOM cohort. Polymorphism rs817826, located between the *RAD23B* and *KLF4* genes, has been identified as being associated with prostate cancer susceptibility^48,49^ though a mechanism is yet to be realized. *KLF4* encodes a zinc finger protein that has been identified to be critical in neural stem cell differentiation^50^ and the regulation of axon growth^51^. *KLF4* has been implicated in the regulation of neuroinflammation^52,53^, and specifically neuroinflammation and oxidative stress in Alzheimer’s disease (AD)^54^, both processes affecting memory performance. Consequently, the role of rs817826 in neurological function warrants further investigation.

Interrogation of the substructure of the cognitive measures was accomplished using PCA. Data reduction via PCA is widely used in association studies and allows for comprehensive analysis of correlated phenotypes^12,13^, though can only be applied when all phenotypes are measured for all samples^55^. In this study, an increase in the number of significant loci was gained when using the multi-phenotype PCA based approach, and, interestingly, none of these loci passed the genome wide significance threshold in any individual cognitive measure. One of the most notable findings from this analysis was the identification of multiple variants in the region of Thiamine Transporter 2 gene *SLC19A3* (rs12105620, rs7578840, rs7579047); rs12105620 in the 3’ UTR being associated with the first cognitive PC that features high loading of visual and verbal learning scores. *SLC19A3* encodes a transmembrane thiamine (vitamin B_1_) transporter that is ubiquitously expressed, and primarily located in the basement membrane and perivascular cells in cerebral blood vessels^56^. Pathogenic variants in this gene have been implicated in basal ganglia disease and encephalopathy^57–59^, and disrupted thiamine transport linked to AD pathogenesis^60,61^. Thiamine deficiency impairs learning ability associated with a related impaired hippocampal neurogenesis in mice models^62^, and plays a major role in alcohol dependent neurocognitive disorders^63^. Low levels of thiamine metabolites have also been associated with decreased Mini Mental State Examination (MMSE) Recall score^64^. These findings support the association between thiamine levels and metabolism and cognition, and further research into the role of *SLC19A3* in neuropathology and cognition is warranted.

In addition to these results, associations were observed between the first cognitive PC and variants in the 3’ UTR of *PPARD* (rs9658167). *PPARD* encodes for a peroxisome proliferator-activated receptor (PPARδ), from a class of proteins which play a role in numerous metabolic functions^65^, including neurogenesis^66^, and demonstrates widespread localisation in the brain and central nervous system, particularly in the hippocampus and hypothalamus^67^. *PPARD* has been implicated in schizophrenia^68^ and major depressive disorder (MDD)^69^, with studies in mice identifying a role for PPARδ in the proliferation and differentiation of neuronal stem cells in the hippocampus^70,71^ and knockout studies demonstrating loss of neuronal and synaptic structure leading to memory dysfunction^72^. A recent cohort study by Insel et al. identified variant rs71567499, approximately 20 kb downstream from rs9658167 and exon 8 of the *PPARD* gene, as being associated with lower Preclinical Alzheimer Cognitive Composite scores in an asymptomatic pre-clinical AD cohort^73^. The effect size of the rs71567499 variant reported in Insel et al. is similar to the effect size observed for rs9658167 in the GRC GOM cohort (β= −2.16 and β= −2.99, respectively) supporting the findings of both studies and the potential role of *PPARD* in memory and cognition.

Also associated with cognitive PC1 is a variant in an intron of *RBFOX1* (rs17138790). *RBFOX1* encodes for an RNA binding protein and splicing factor that regulates the expression of numerous genes involved in neuronal and synaptic development^74,75^. Meta-analyses of 232,964 cases and 494,162 controls by the Cross-Disorder Group of the Psychiatric Genomics Consortium recently identified intronic variant rs7193263 in *RBFOX1*, (400 kb downstream from rs17138790), as a pleiotropic locus being associated with 7 neuropsychiatric disorders, including autism spectrum disorder (ASD), schizophrenia, bipolar disorder, MDD, and attention-deficit hyperactivity disorder (ADHD)^76^. Lahti et al. (2022) identified a suggestive association between intronic variant rs10852681 in *RBFOX1* and verbal short-term memory in a sample of 44,874 individuals^3^. Variants in *RBFOX1* have also been associated with amyloidosis and reduced cognitive performance in AD^77^. A recent study by O’Leary et al. (2022) demonstrated an increase in neural reactivity to emotional stimuli, captured using functional MRI, as well as reduced prefrontal activation during cognitive control, in participants carrying variant rs6500744^78^, located 200 kb downstream from rs17138790 identified in this current study. Another recent study identified five significant SNPs in the *RBFOX1* gene in close proximity and in high LD with lead variant rs75885813, as being significantly associated with working memory impairment in a juvenile ADHD cohort^79^. Visual memory was assessed in the study using the Rey–Osterrieth Complex Figure (ROCF)^80^, which involves copying a complex geometric shape, at a 20-minute delay. This component is similar to the SVLT DRI, also requiring the recognition of visual compositions following a 20-minute delay; consequently, although utilising different cognitive tests, this reinforces the findings of this current study and further implicates variants in the *RBFOX1* locus as having a role in visual learning and memory.

Additional variants were identified to be associated with cognitive PC2, featuring high loading of prospective and retrospective memory scores. One of these was a synonymous variant in *NXPE3* (rs2305990), a gene encoding for a neurexophilin glycoprotein that is ubiquitously expressed in the brain^81^ and is involved in axonal adhesion^82^. *NXPE3* is yet to be comprehensively investigated, though it has been implicated in adenocarcinoma of the lung^83^. An intronic variant in the *RD3* (rs17189035) gene was also associated with cognitive PC2, and the encoded protein Retinal Degeneration Protein 3 has recently been reported to be localised to the retina^84^. Retinal Degeneration Protein 3 is involved in photoreceptor function^85,86^ and mutations in *RD3* can lead to Leber congenital amaurosis, a disease that causes retinal degeneration and blindness^87,88^. It could be hypothesized that the association of *RD3* variants with the cognitive phenotype identified in this study may be a result of its endogenous role in photoreceptor cells and retinal function, and subsequent impact on cognitive assessment, however further investigation is needed. Finally, an intronic variant rs17130484 was identified in the *WLS/GNG12-AS1* locus, encoding the Wntless Wnt Ligand Secretion Mediator protein, an integral membrane transporter protein that mediates Wnt secretion^89^. Variants in *WLS* have been implicated in structural birth defects^90^, and Wnt signalling has been associated with hippocampal synaptic plasticity^91^ and memory^92^ in mouse models. Dysfunction of Wnt signalling has been implicated in neurological disorders including AD^93,94^; however, *WLS* is yet to be associated with cognitive function and its role in prospective and retrospective memory should be further explored.

Replication of suggestive associations in the GRC GOM cohort was performed in two cohorts, one a subset of the QIMR Twin cohort^32^ and the second the GOBS study cohort^33,34^. Variant rs9948718 was identified to nominally correlate with working memory in the GRC GOM and QIMR Twin cohorts and is located downstream of *MAPK4*. *MAPK4* is yet to be linked to memory or cognition, although has been reported as being overexpressed in gliomas^95^. Variants rs1032704 and rs4931672, upstream from *PKP2*, were identified to be suggestively significantly associated with visual learning in the GRC GOM cohort, and nominally associated with visual memory in the GOBS cohort. These two variants are also yet to be associated with memory or cognition, however, upregulation of *PKP2* has been associated with the progression of gliomas^96^, suggesting potential congruities between variants rs9948718, rs1032704, and rs4931672 that may warrant further investigation. Variant rs6035340, an intronic variant in *SLC24A3*, was suggestively significant in the GRC GOM cohort for MIST DELAY and replicated in the GOBS cohort for CVLT Delay^47^, associating this variant with visual learning and memory. *SLC24A3* encodes a potassium-dependent sodium/calcium exchanger, highly expressed in the brain, and has been associated with abnormal motor learning, but not cognitive function, in mice^97^, and as a risk loci for migraine via GWAS^98^, implicating this gene in neurological phenotypes.

While specific variants identified as being significantly associated with cognitive phenotypes in our analysis have not been previously reported in similar studies, the genome-wide significant variants identified in this study map to several genes reported in previous cognitive GWAS. Variant rs10889966 is located upstream of *NEGR1*, which has been previously associated with cognitive performance in a previous GWAS^99^. *NEGR1* has also been reported to be associated with MDD^100,101^ and AD^102,103^, with its role in neuronal development, cognition, and learning widely studied in murine models^104–106^. Similarly, variant rs17130484, significantly associated with cognitive PC2, is located in *WLS GNG12-AS1* which has been associated with cognitive processing speed^107^, while rs17189035, located in *RD3* and also significantly associated with cognitive PC2 was been previously associated with intelligence^108^. Differences in the cognitive phenotypes analysed between our study and similar studies, as well as the diversity of ancestry in our cohort, may both contribute to the novelty of our findings; however, these associations suggest convergent evidence at the gene level for our findings when compared to similar studies, despite variant-level novelty.

Pathway analysis using SNP sets identified the enrichment of odorant binding pathways in the WASI IQ, HVLT Delay and Retention, PRMQ RM, and SVLT RII subsets, implicating olfactory factors in the intelligence, verbal learning and memory, and retrospective memory domains. Enrichment of taste receptor pathways was also identified in HVLT Delay measures, specifically sensory perception of bitter taste. The association between odour and memory has been extensively explored, as reviewed by Saive et al. (2014)^109^ and Tong et al. (2014)^110^, with patterns of olfactory and verbally cued memory and associated brain activation identified in the evocation of autobiographical memory^111^. In addition, olfactory factors have been identified as being potential biomarkers for cognitive impairment with bearing on AD^112,113^, consolidating the associations between olfactory processes and memory. The mechanisms and relationship between taste and memory has been well documented^114^, with the association between taste perception and working memory also being reported^115^, in line with the findings in this study.

Enrichment of cadherin pathways were also identified in significant SNP sets in the HVLT discrimination and PRMQ RM measures representing verbal learning and retrospective memory, respectively. Cadherins are well understood to be critical in neuronal and synaptic architecture^116,117^, and have been functionally implicated in working memory and learning using mouse models^118,119^. Cadherins have been associated with working memory performance in an ADHD cohort^120^, and with neurocognitive disorders including ASD^121,122^, as well as being implicated in Aβ production and amyloidosis^123,124^. Further investigation into the role of the cadherin family of proteins in memory and cognition is justified.

Flavonoid and uronic acid metabolic processes were over enriched in the PRMQ PM, HVLT Learning, CAPM IADL, and WAIS LNST subsets, implicating these pathways in prospective memory, verbal learning, and working memory. Enrichment of these pathways in these sets are a result of several suggestively significant variants within a complex locus of uridine 5’-diphospho-glucuronosyltransferase (UGT) genes. UGTs are not ubiquitously expressed in the human brain; although, *UGT1A4* has been reported to be expressed in endothelial and neuronal cells^125^. Glucuronate has also been associated with schizophrenia^126^, while uronic acid pathways have been implicated in AD and amyloidosis^127^. In a similar manner, enrichment of mineral ion metabolic processes in the CAPM Total test gene-sets are a result of a nominally significant 3’ UTR variant in Metallothionein 1 M gene *MT1M*, located in a multigenic region and in the vicinity of several other metallothionein genes. Enrichment of pathways in this manner is a limitation of the set-based method, and could be considered a trade-off when compared to other methods, such as SNP ratio tests, which cannot identify significant pathways that may contain multiple smaller effect loci and are unfit for unimputed or lower coverage data^128,129^.

Enrichment of the retinol metabolic process pathway in cognitive PC1 was also reported, this pathway being distinct from those associated with UGT genes. A-vitamins, namely retinol and its metabolite retinoic acid, are well established as being integral in normal cognitive function and neuroplasticity^130,131^. Contemporary studies have implicated vitamin A in ASD^132^, AD^133–135^, and cognitive function in later life^136,137^. These findings, notably the enrichment of retinol metabolic pathways for cognitive PC1 with high loading of visual and verbal learning scores, reinforces the proposition of a genetic link between cognition and vitamin metabolism that should be further explored.

### Limitations

The small sample size is a limitation for this study when compared to large scale GWAS. Imputation was not considered due to the multi-demographic nature of the cohort and may have improved coverage. Specific significant variants identified in our current study were not replicated in similar cognitive GWAS. This is likely due to ethnicity differences in our cohorts that are not widely studied, or reported in similar studies, as well as differences in the cognitive phenotypes assessed in this study compared to similar studies. Our interrogation of the genetic substructure of our cohort indicates significant diversity of ancestry which supports this. The pathway analysis method is also sensitive to the overrepresentation of variants in multi-gene regions, and imputation may have allowed for more refined pathway analysis; however, as discussed above, it would have disputable impacts on the sensitivity of the analysis. For some of the findings in this study, there is currently insufficient evidence in the literature to explain the role of the genes identified. For the replication study, a significance level of *P* < 0.05 was used for analysis to avoid overly conservative replication criteria given the small sample size of the replication cohort. Further assessment in similar cohorts and via functional studies would be required to elucidate the mechanisms of the variants and genes implicated in this study.

## Conclusion

Results identified 13 genome-wide significant SNPs and 289 SNPs passing the threshold of suggestive significance across all 24 phenotypes. We found a significant novel association between the rs817826 SNP located between the *RAD23B* and *KLF4* genes and verbal learning discrimination. PCA identified three variants in the vicinity of thiamine transporter gene *SLC19A3*, including one within the 3’ UTR, which were associated with the cognitive PC explained by HVLT and SVLT test batteries that quantify of visual and verbal learning, respectively. This cognitive PC was also associated with a 3’ UTR variant in the *PPARD* gene and an intronic variant in *RBFOX1*. The cognitive PC with loadings dominated by CAPM and PRMQ test batteries, measuring prospective and retrospective memory, was identified to be associated with a synonymous variant in *NXPE3*, as well as intronic variants in *RD3* and *WLS/GNG12-AS1*. Pathway analysis identified olfactory, vitamin A, and cadherin pathways as being notably overrepresented, exhibiting significant associations across multiple cognitive domains. These findings provide novel insights into the association between genetics and cognition in healthy individuals and provides a basis for future research.

## Supplementary Information

Below is the link to the electronic supplementary material.

Supplementary Material 1

Supplementary Material 2

Supplementary Material 3

Supplementary Material 4

Supplementary Material 5

Supplementary Material 6

## Data Availability

The GWAS summary results for all significant and suggestive SNPs are available in Supplementary Data. Full GWAS summary statistics are available in the NHGRI-EBI GWAS catalogue (https://ftp.ebi.ac.uk/pub/databases/gwas/summary_statistics/GCST90448001-GCST90449000/) under accessions GCST90448143-GCST90448166.
