# Supplementary figures and images for: Genome-wide association study identifies novel variants in olfactory, vitamin A, vitamin B, and cadherin pathways associated with learning and memory
Source: Sci Rep. 2025 Dec 18;16:2911. doi: 10.1038/s41598-025-32828-8 (PMC12830837; doi:10.1038/s41598-025-32828-8)

**Supplementary Figure 1:** QQ plots and genomic inflation for test phenotypes.


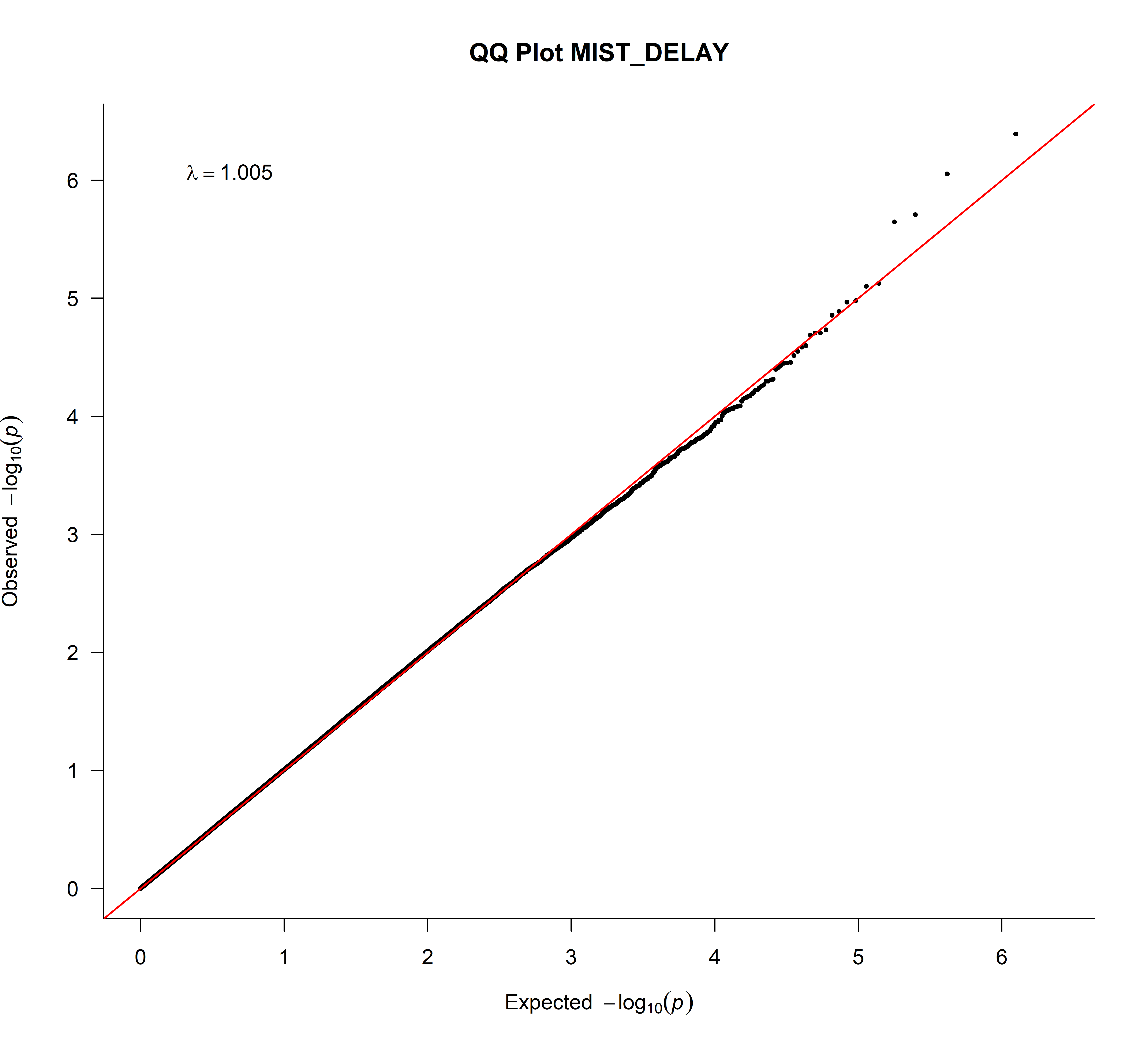

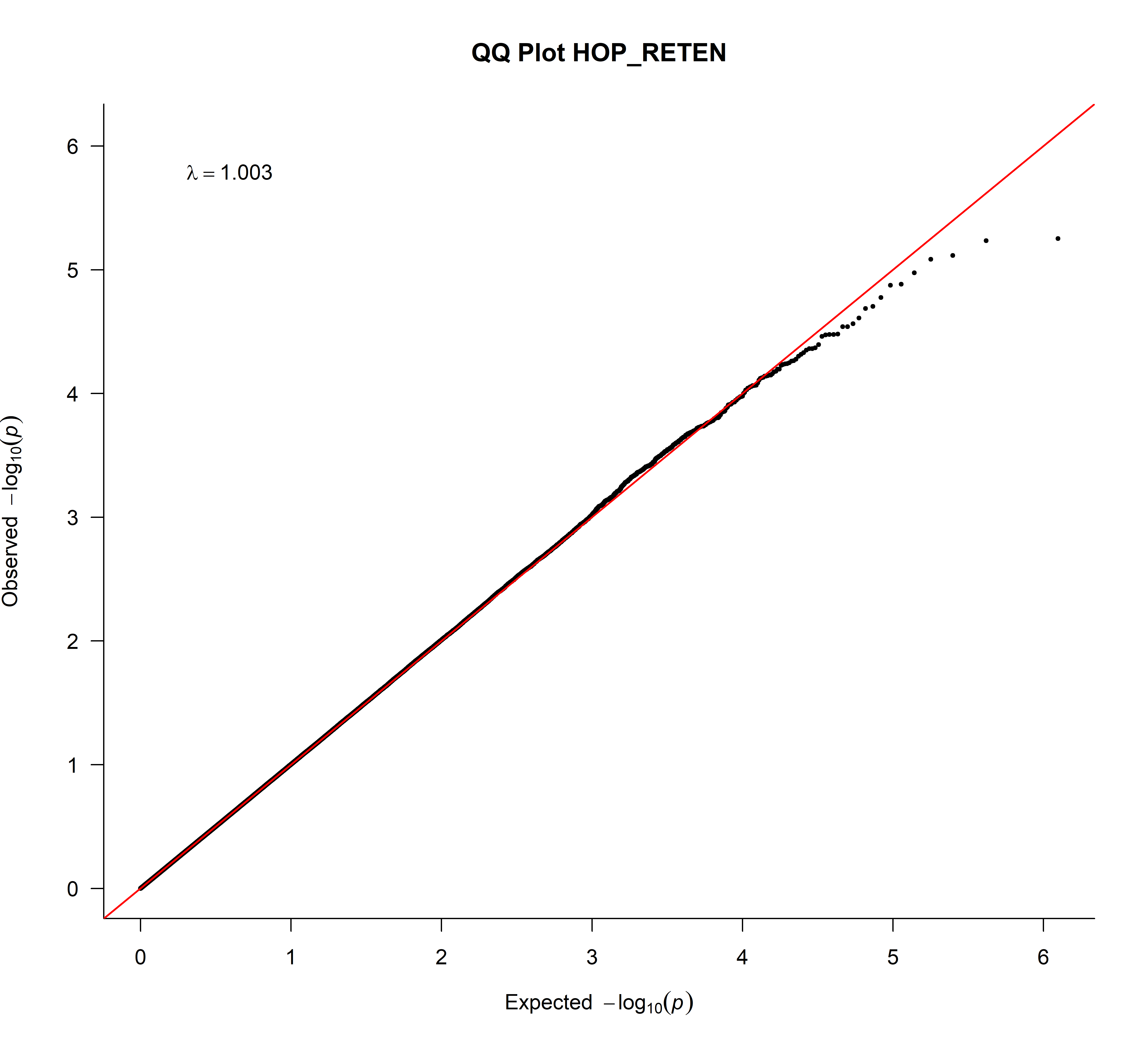

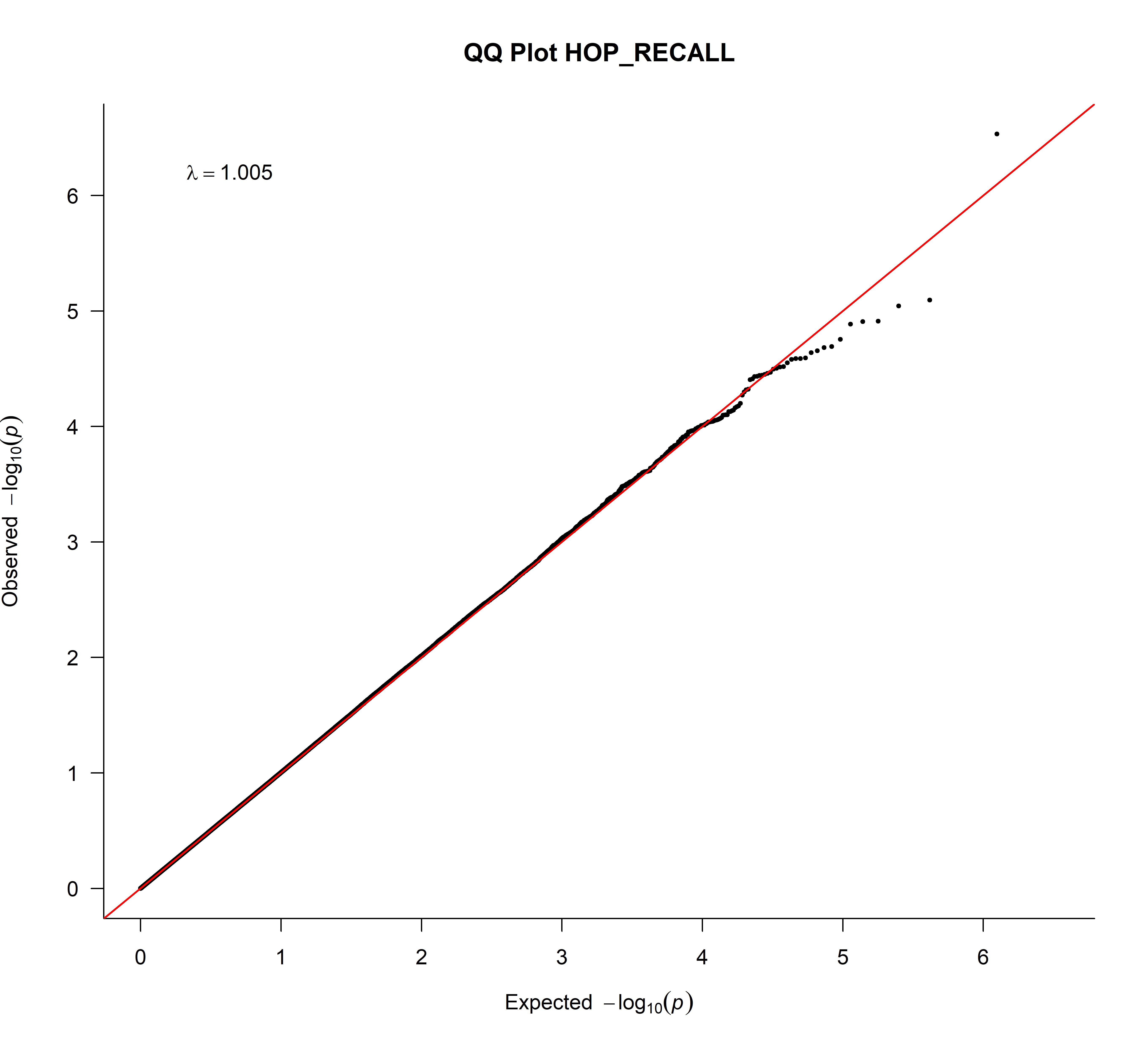

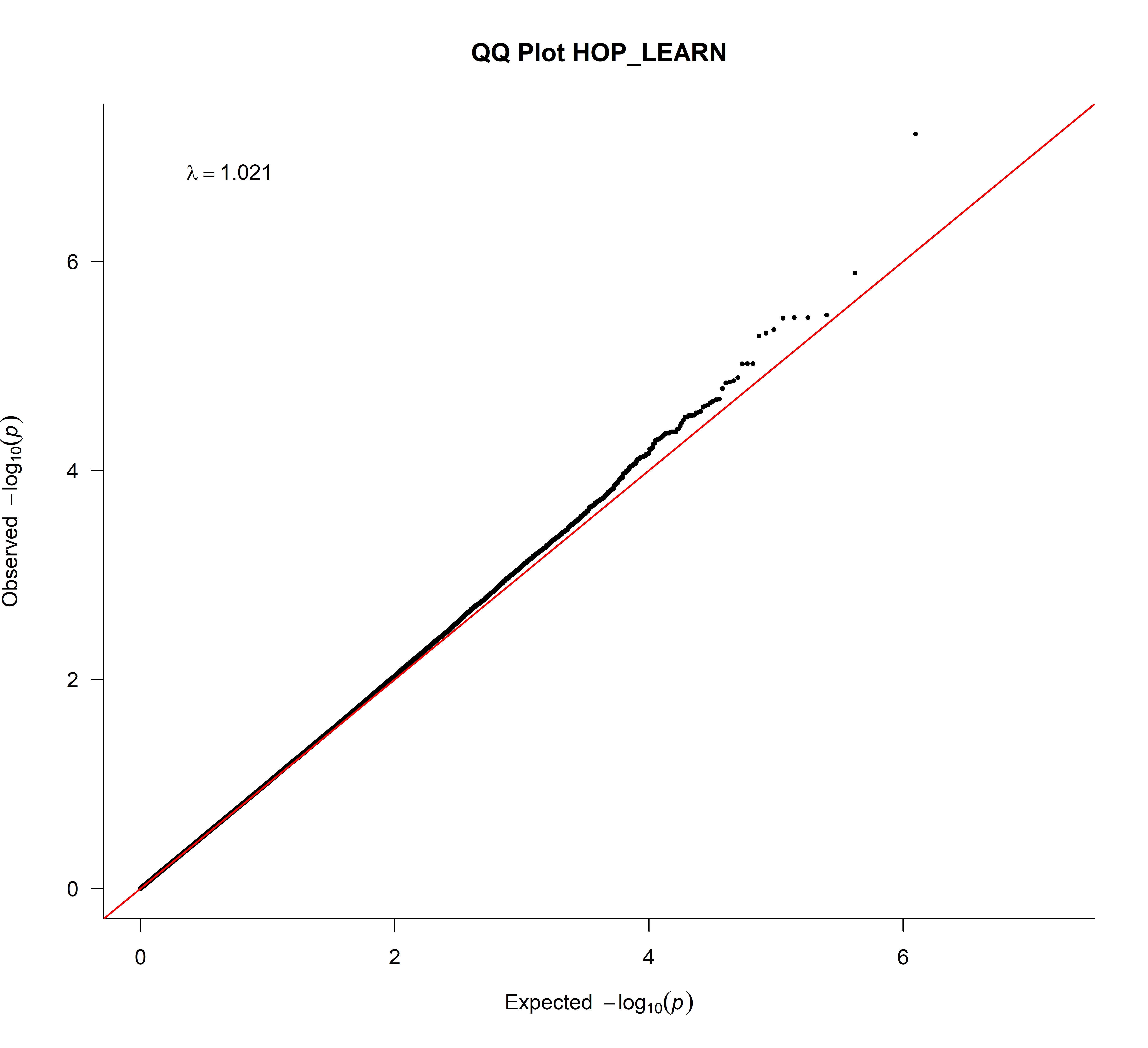

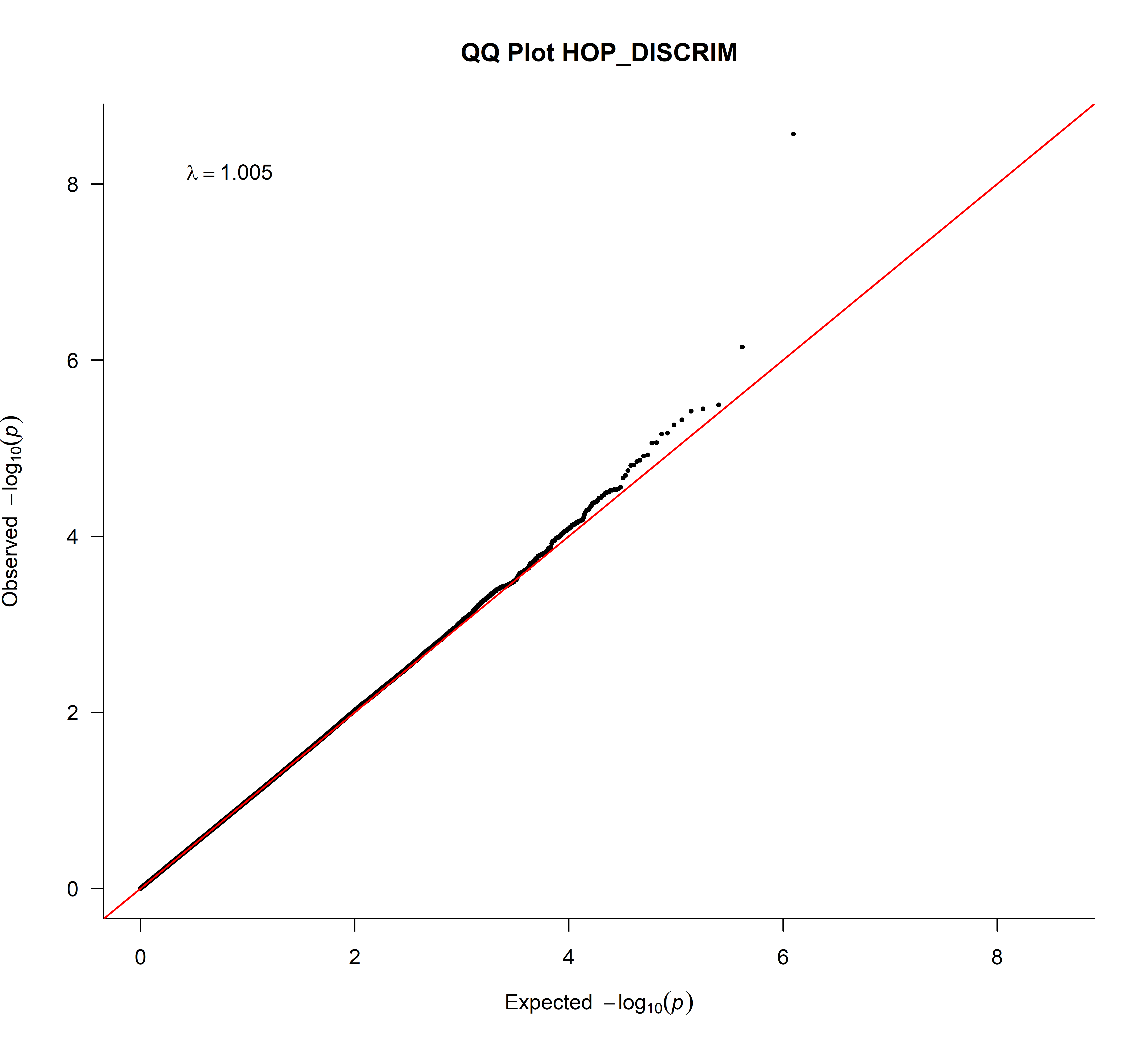

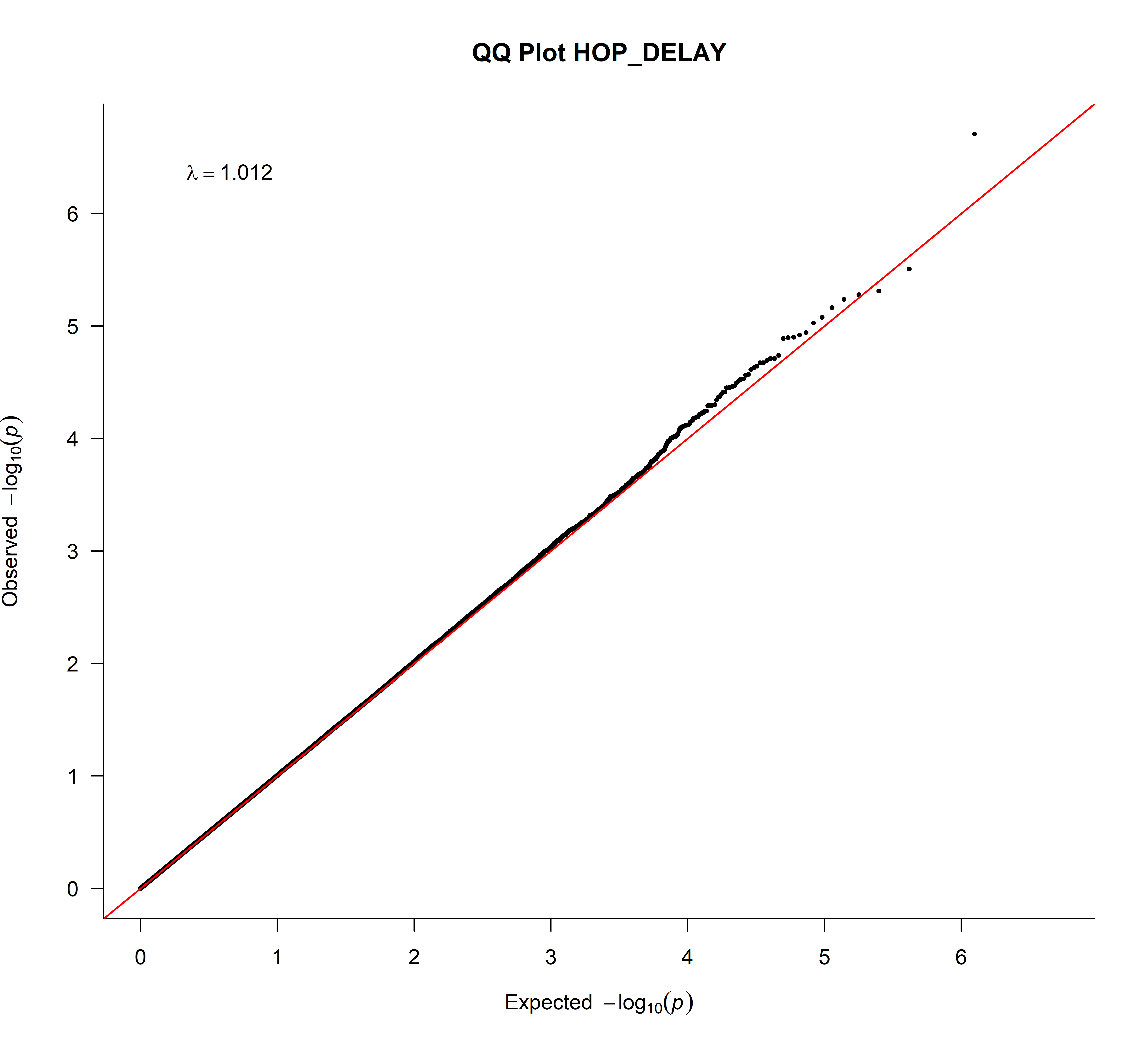

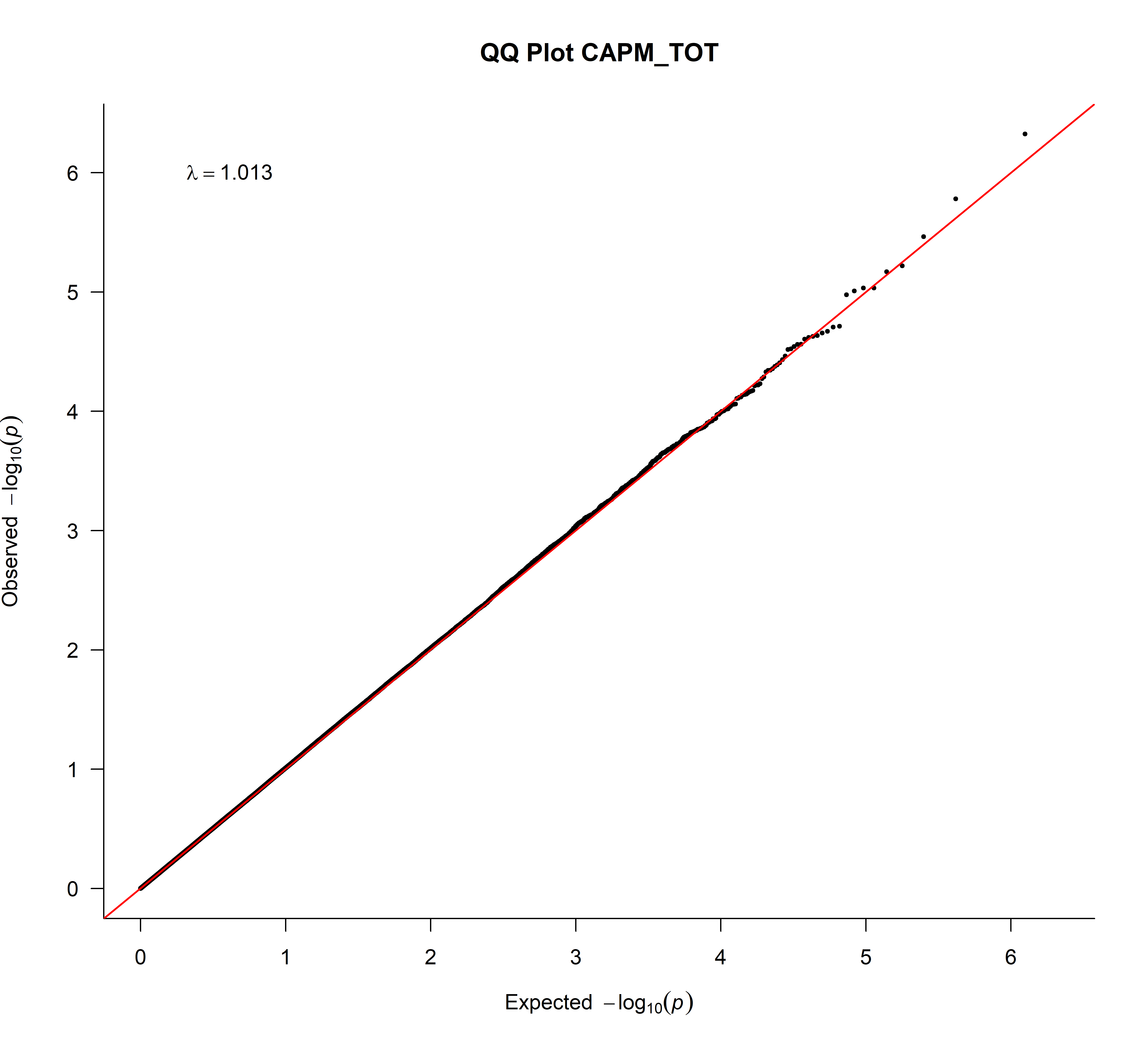

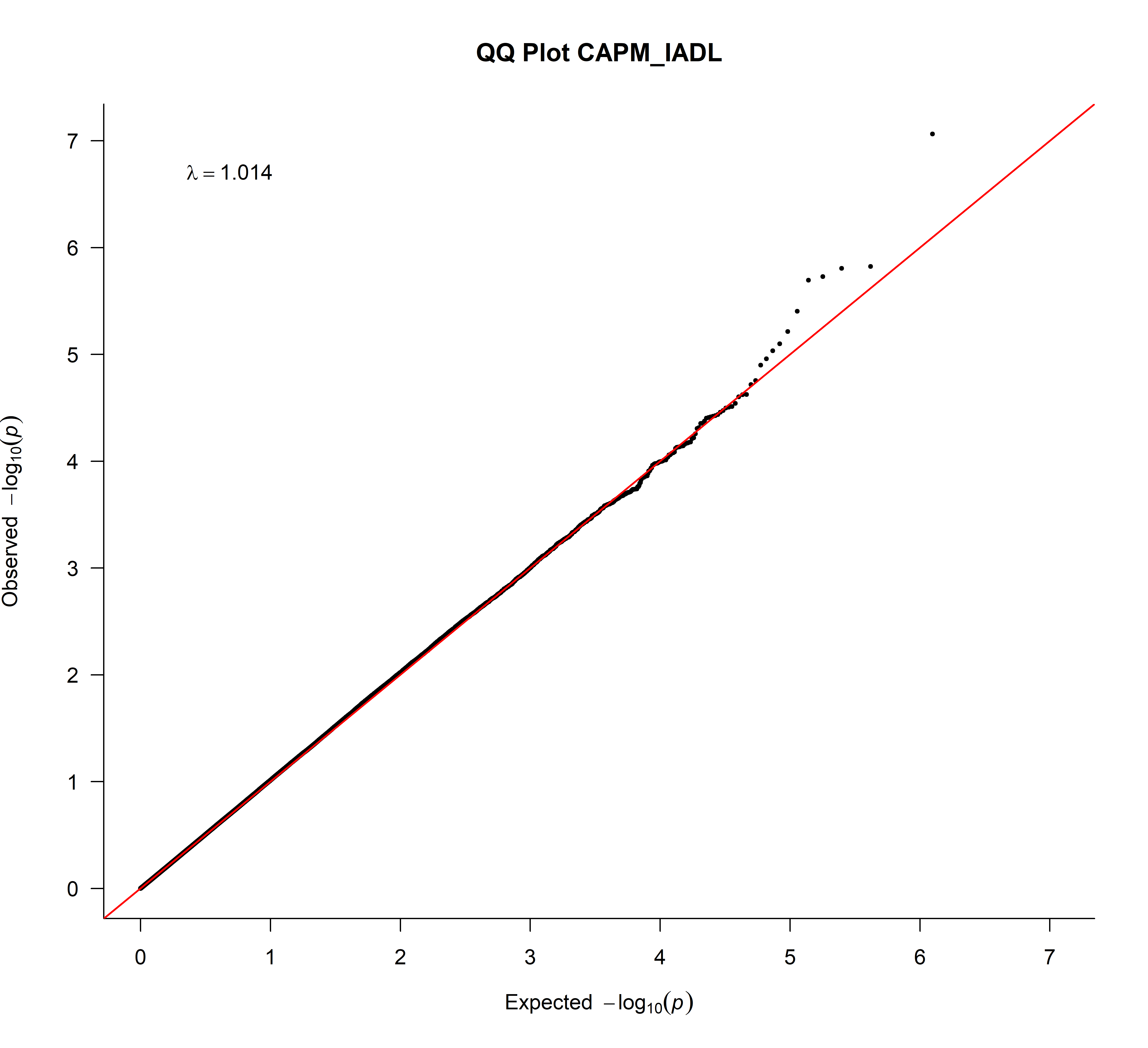

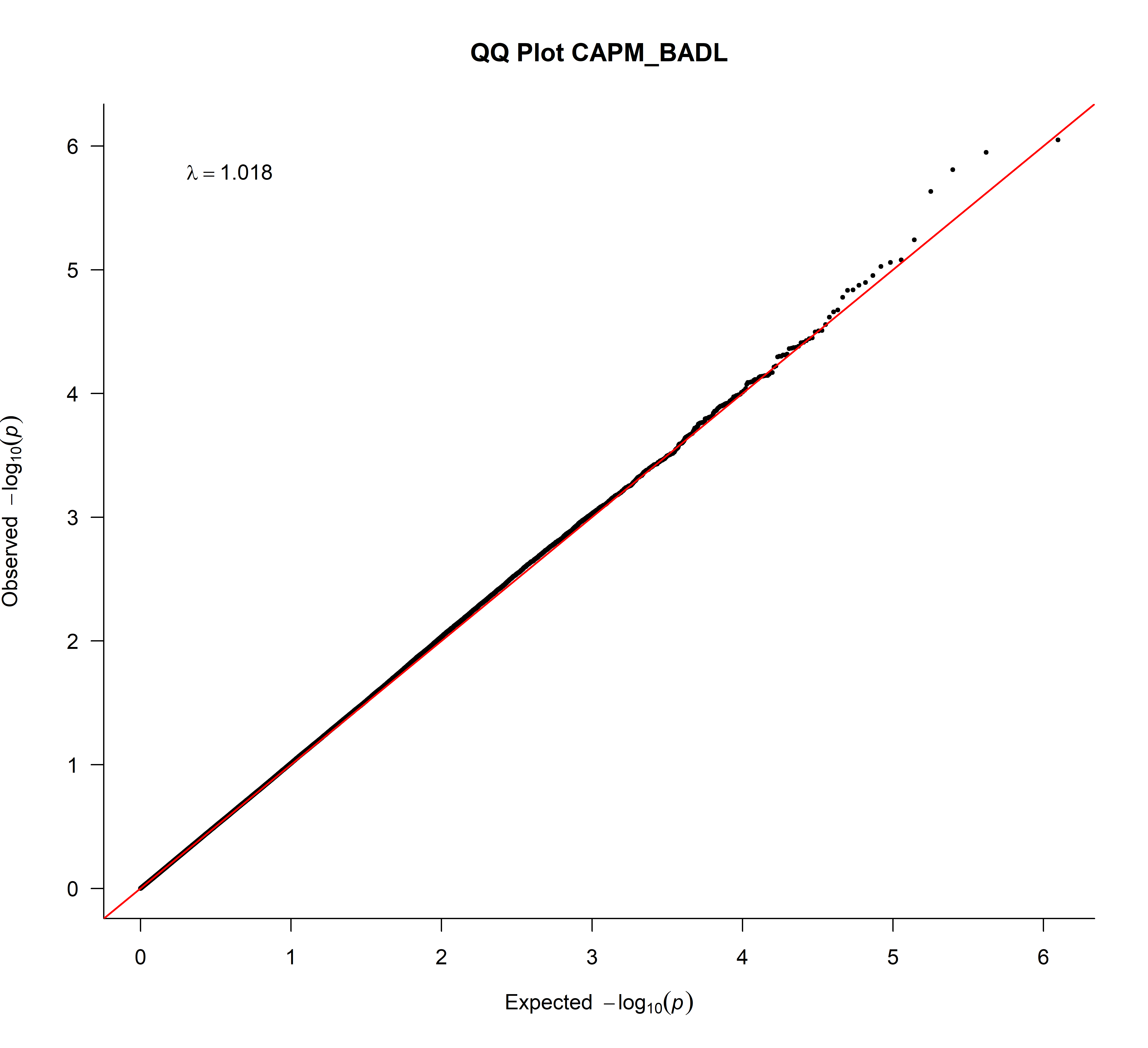

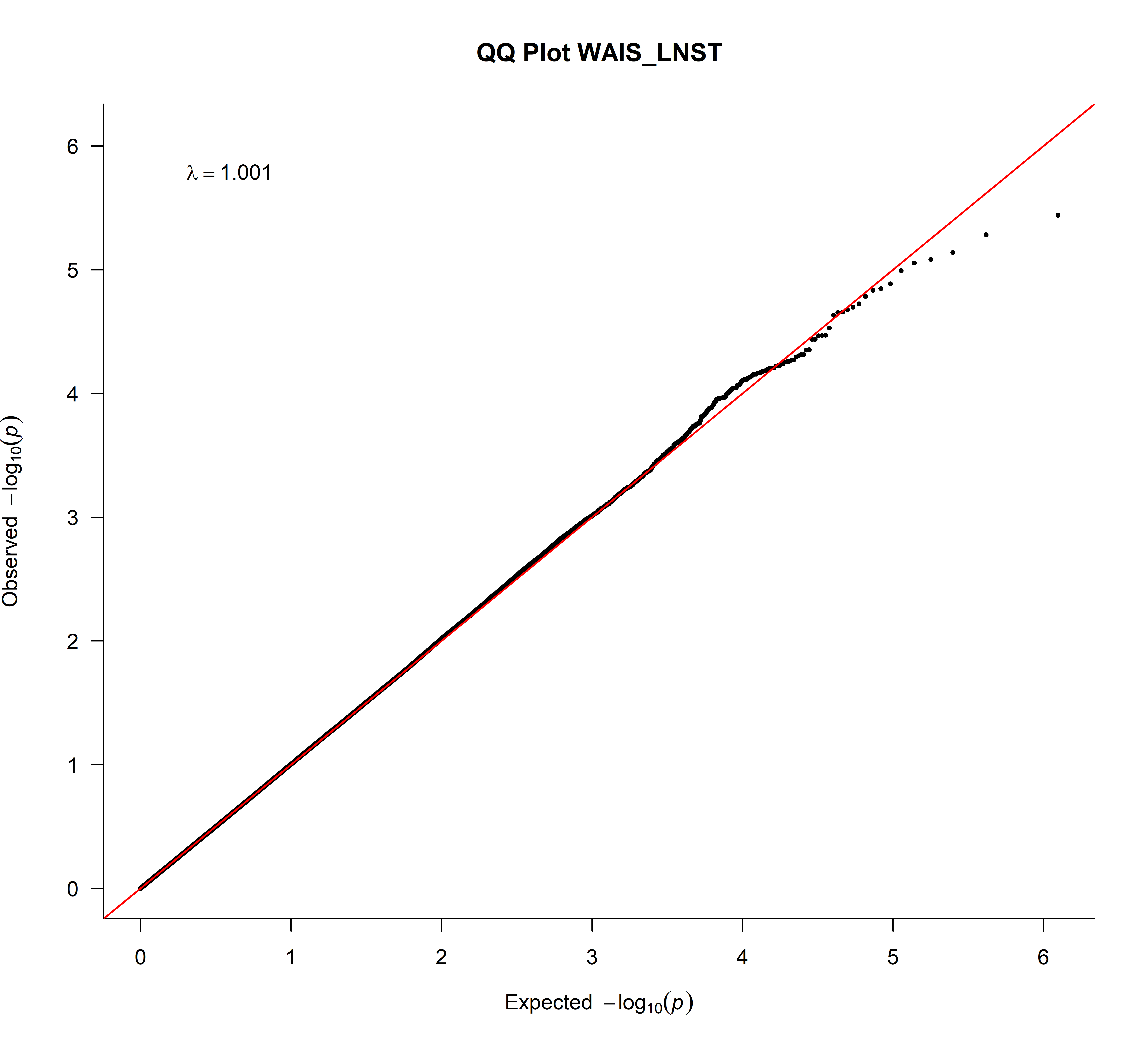

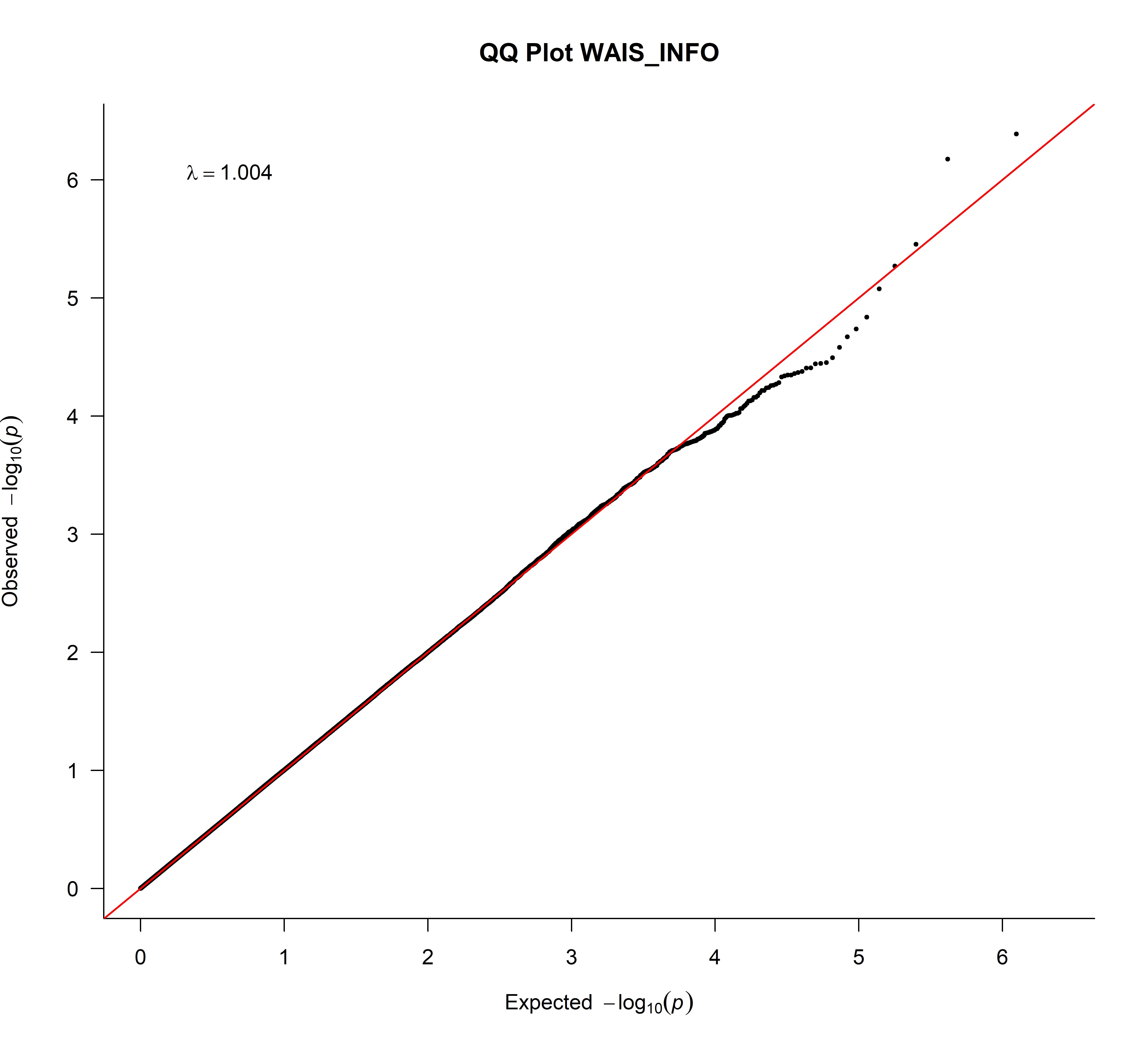

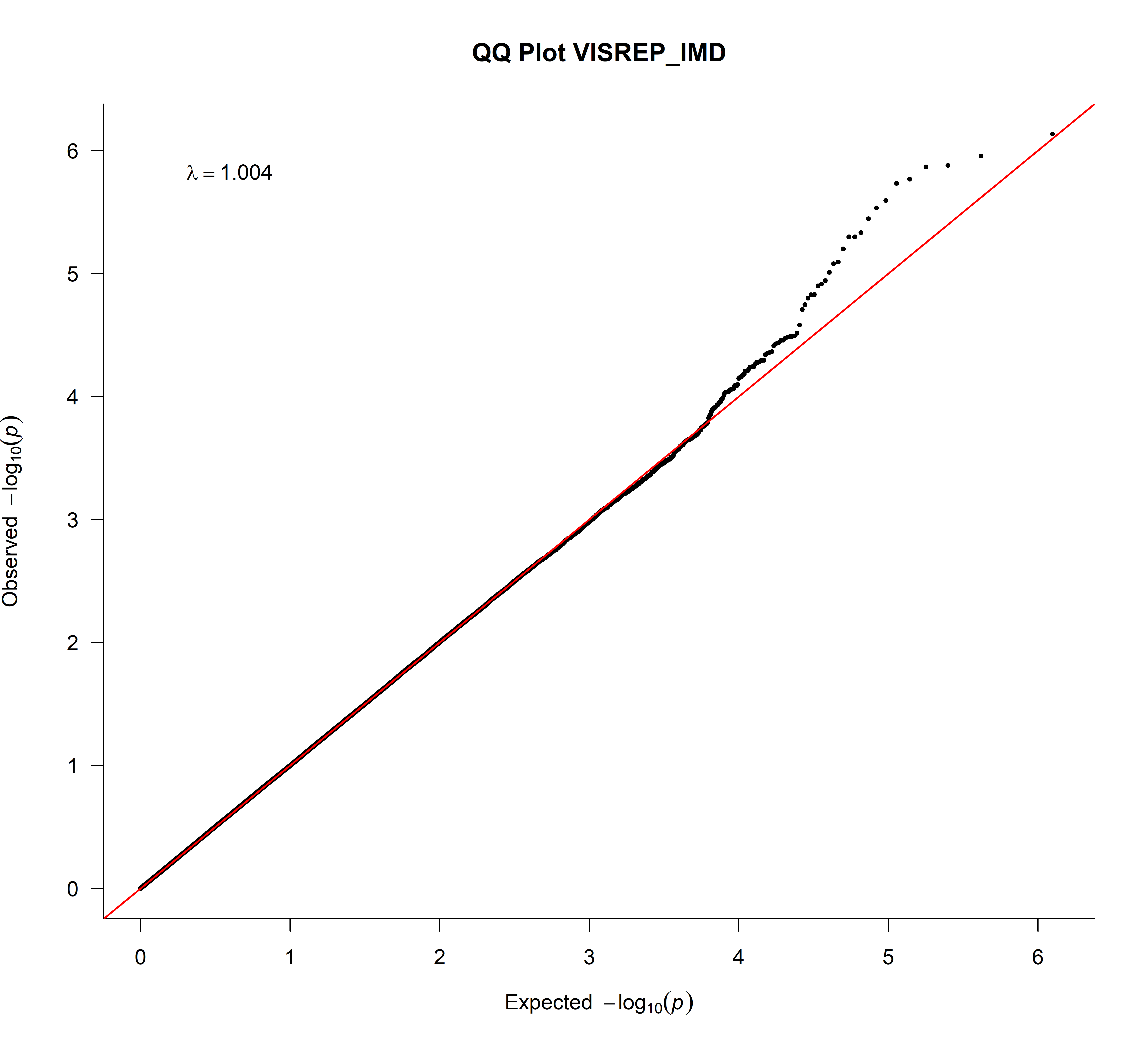

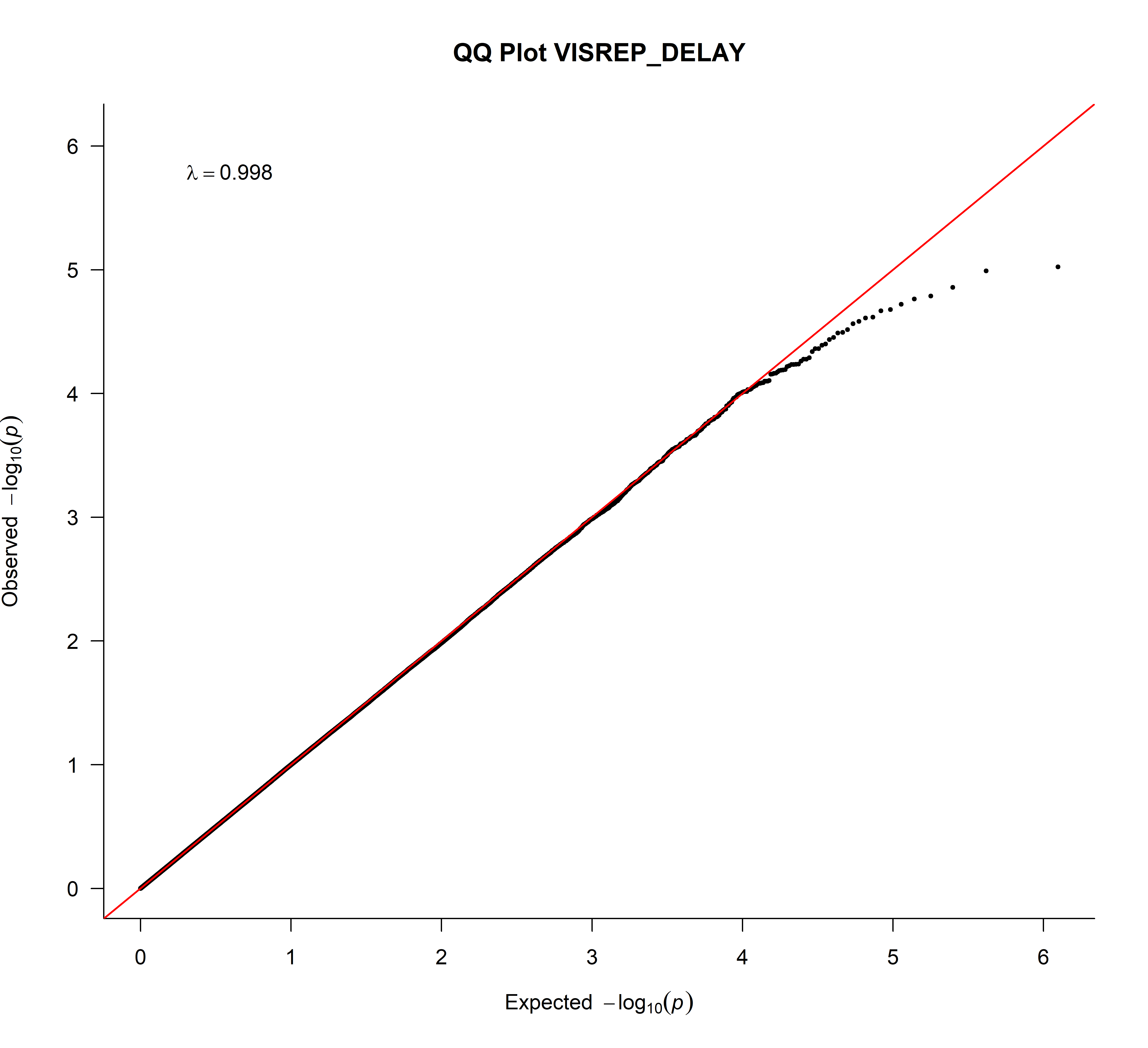

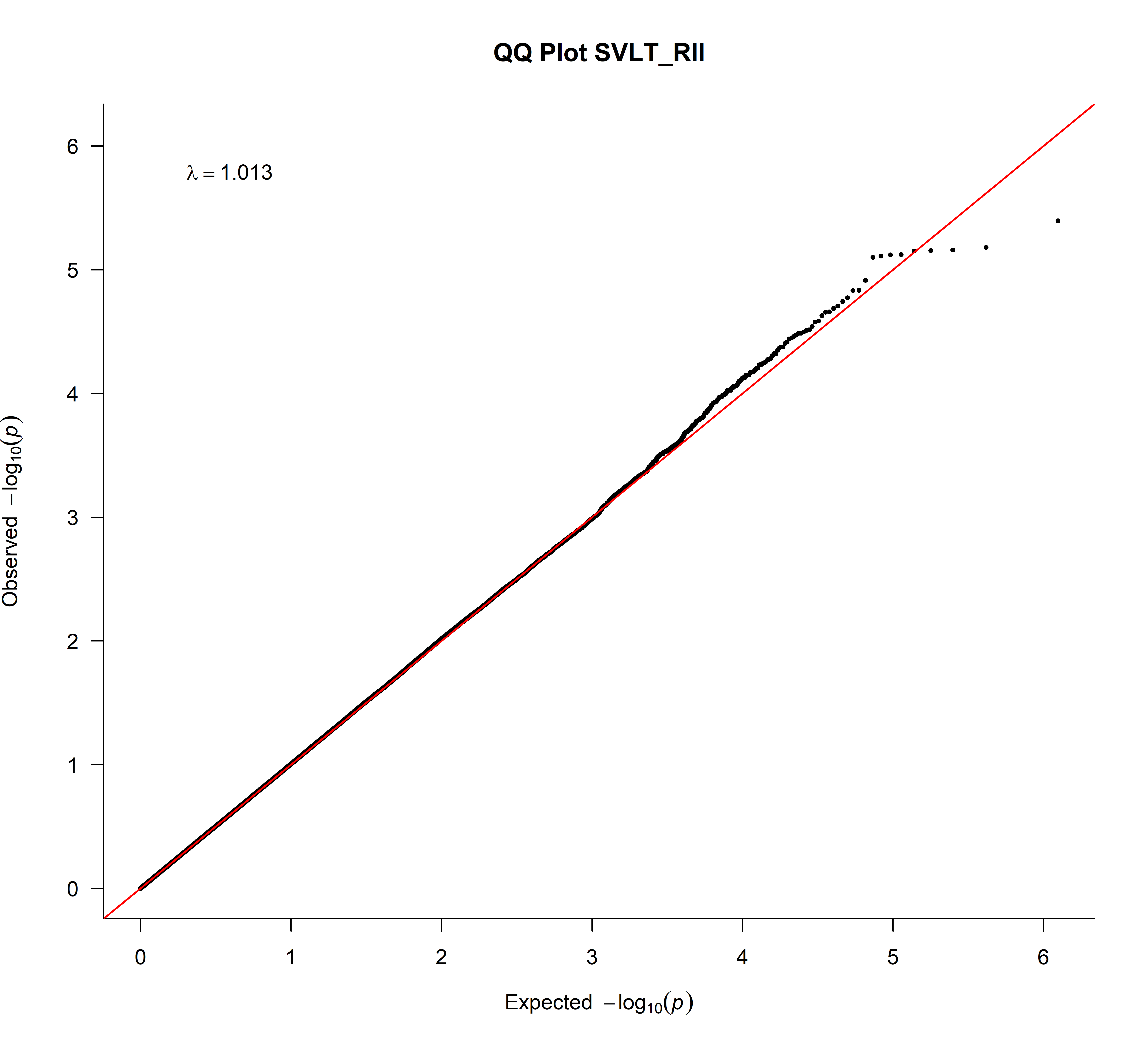

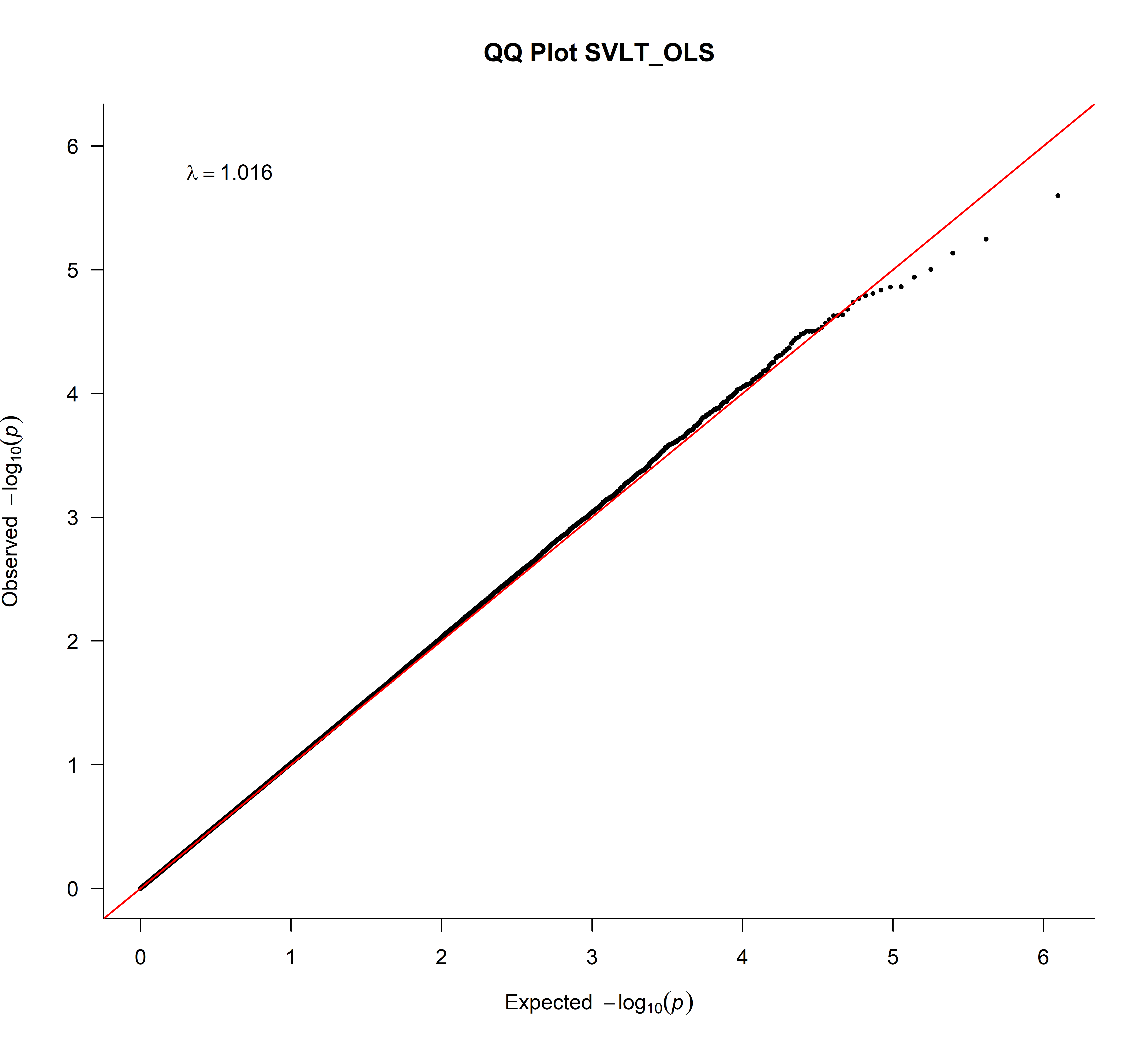

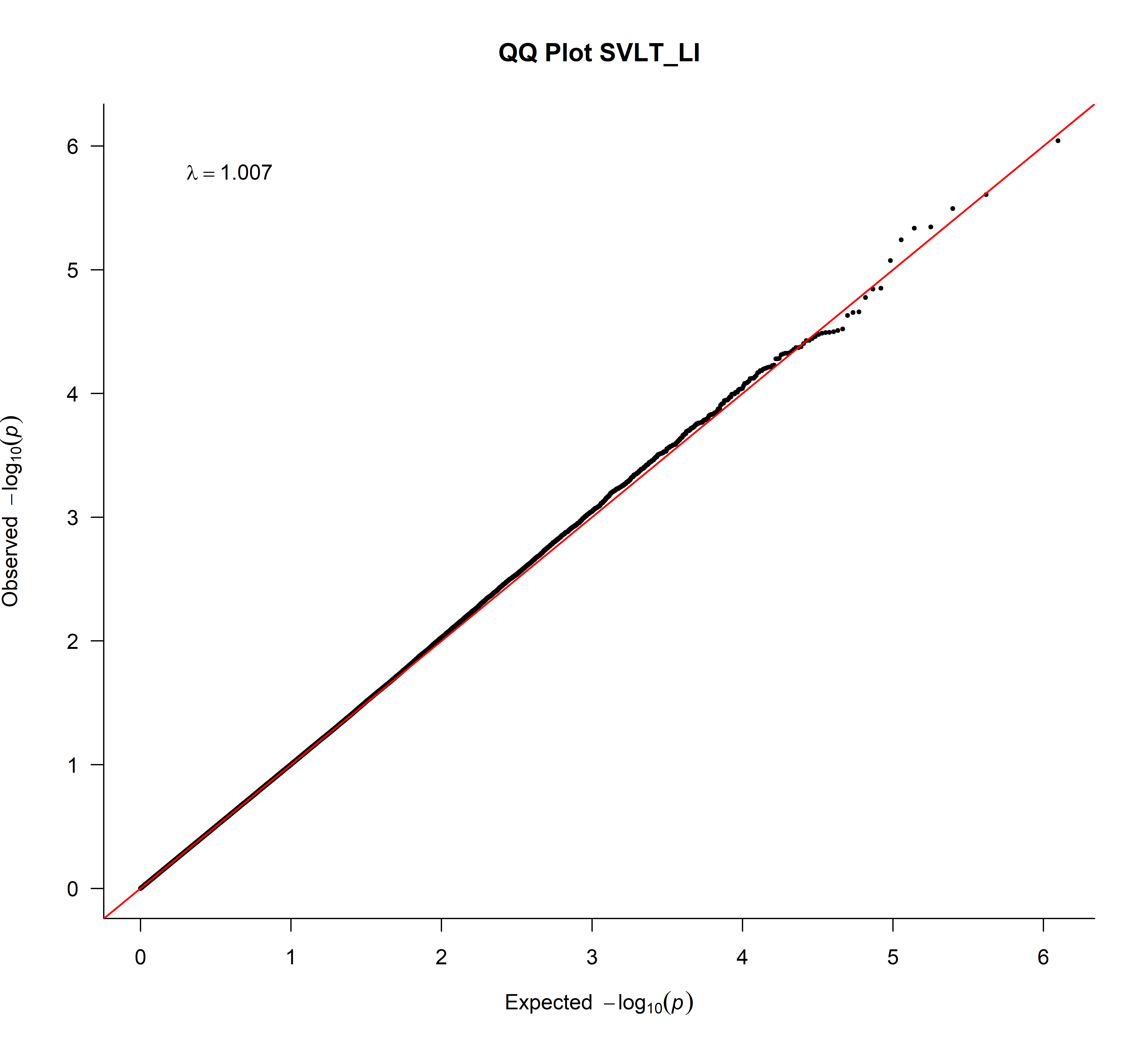

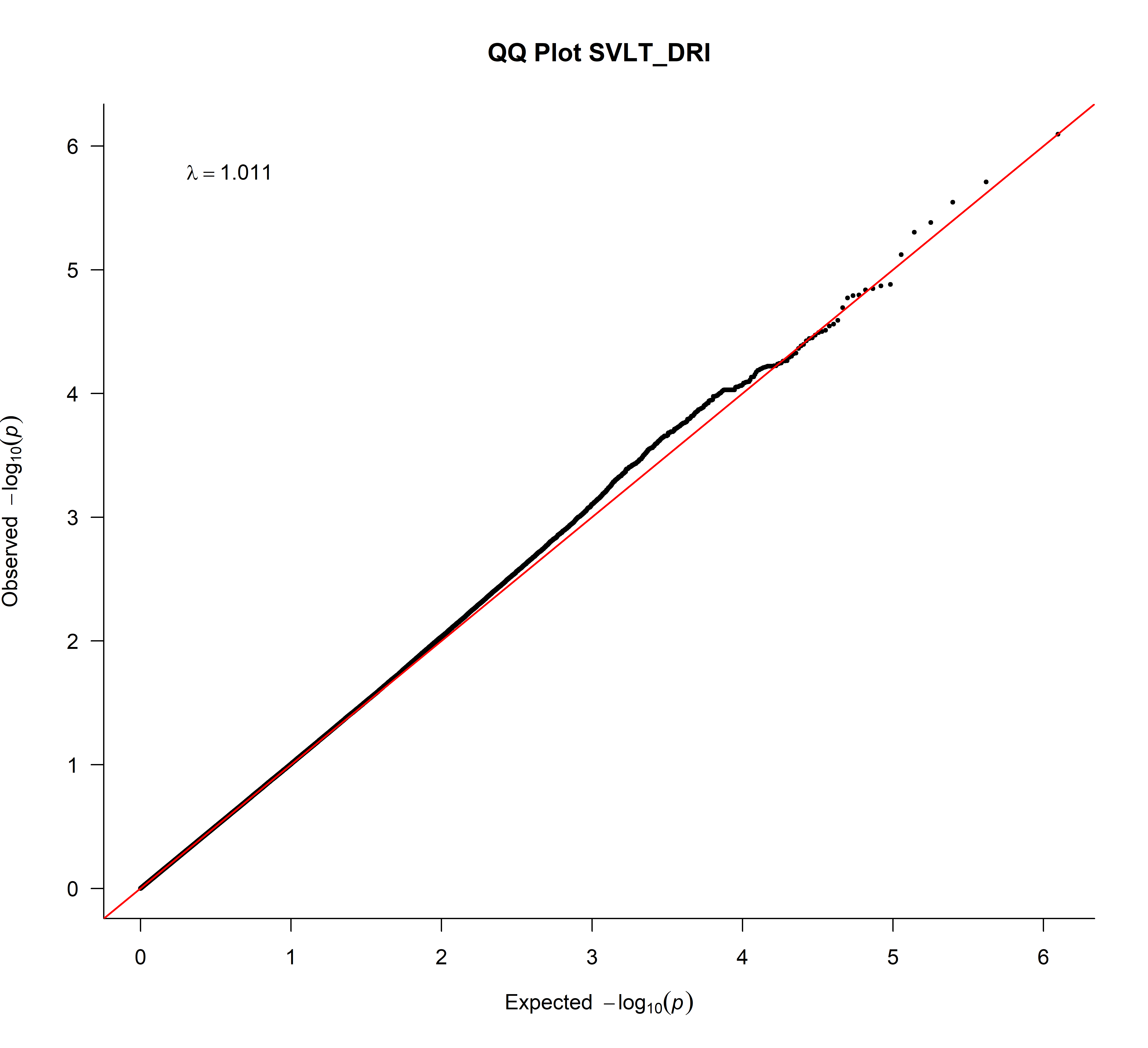

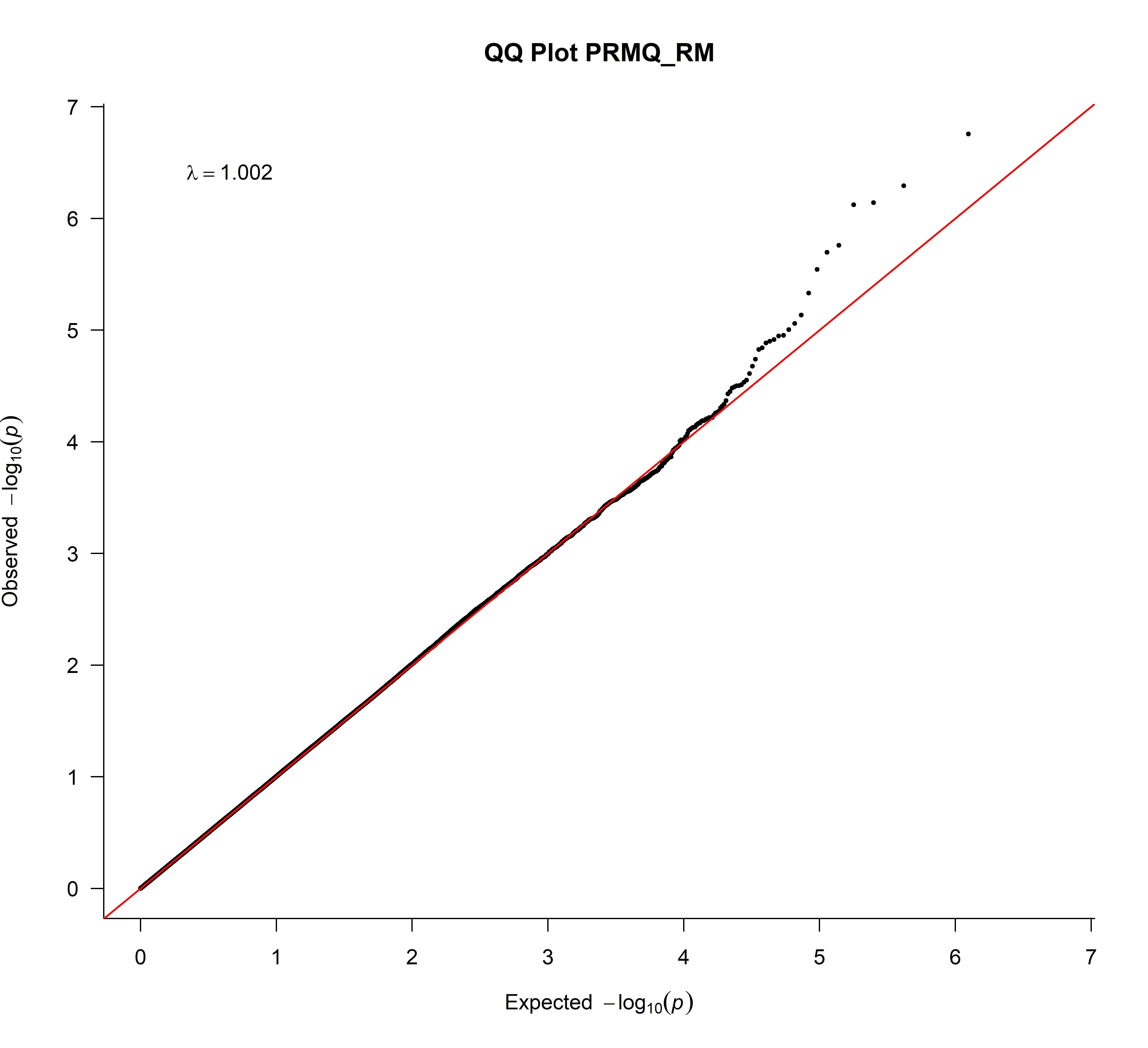

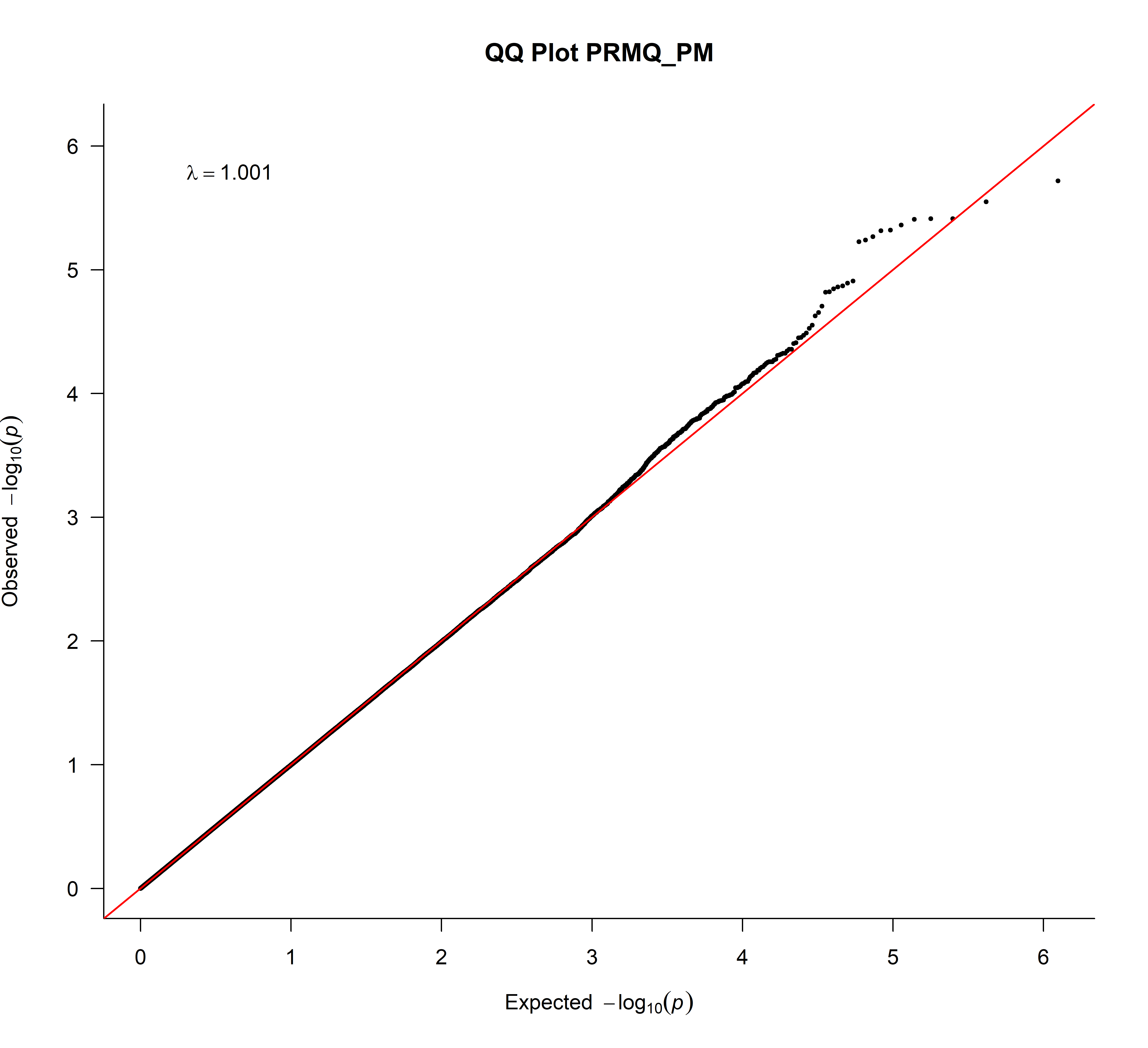

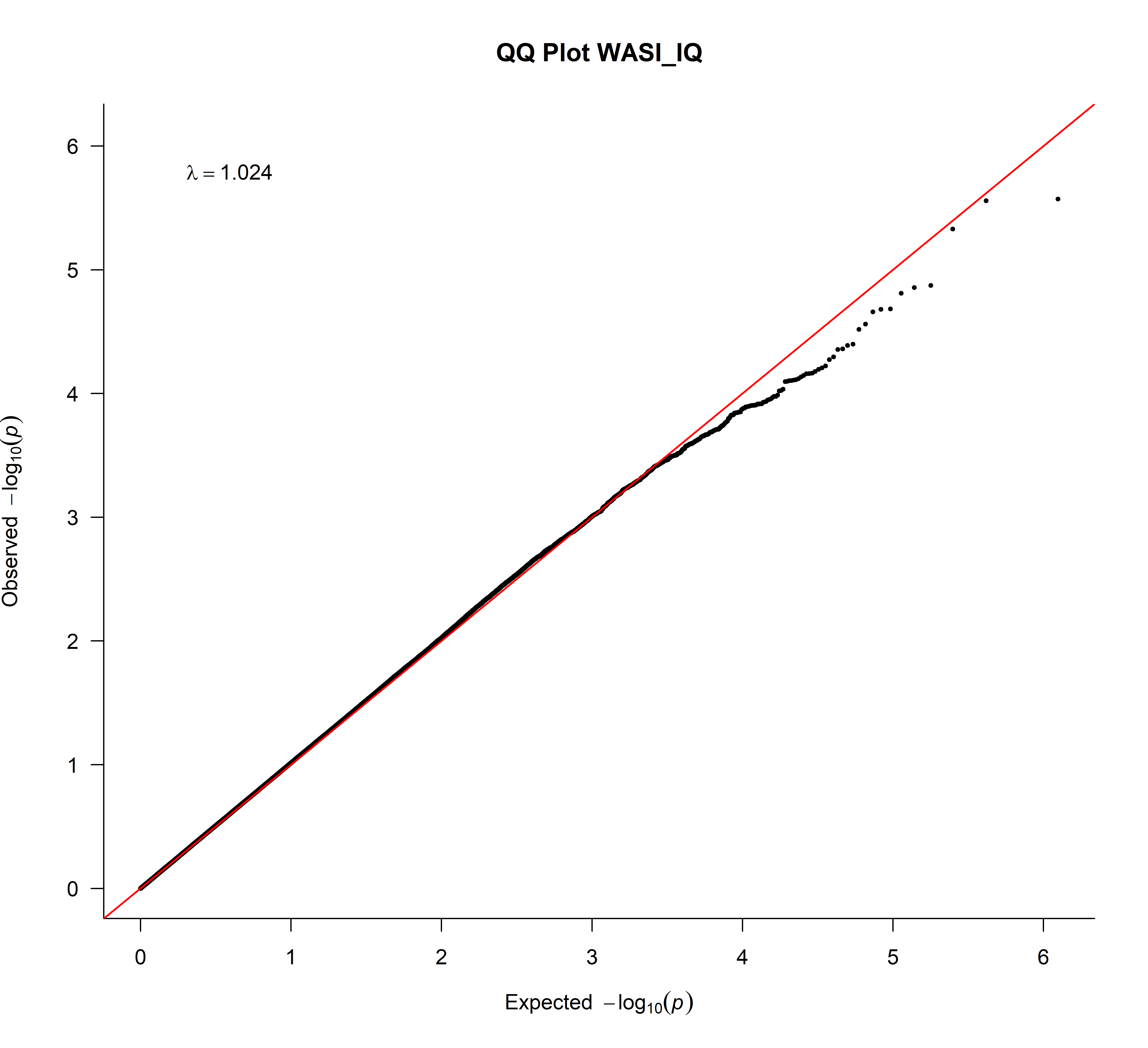

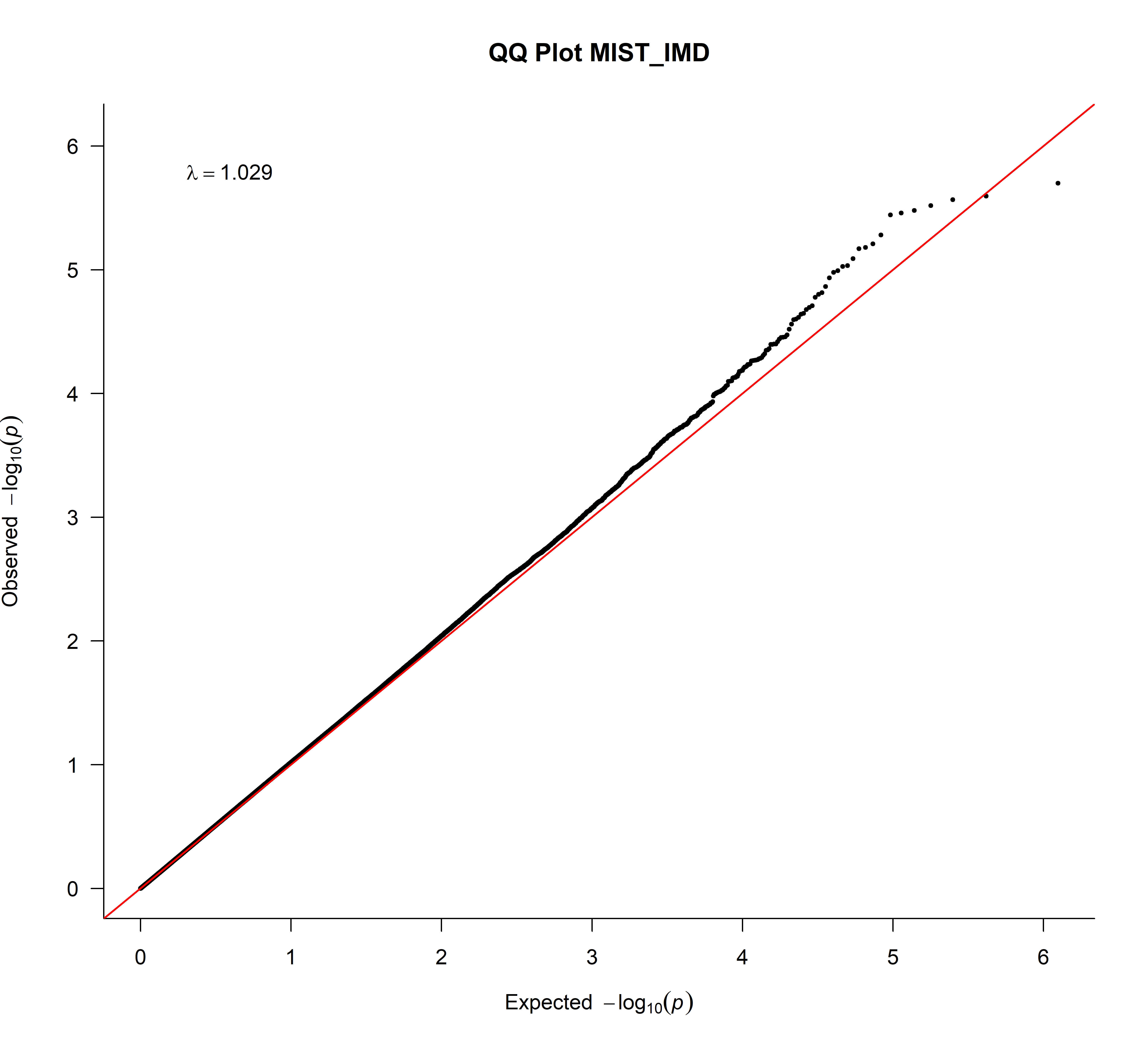

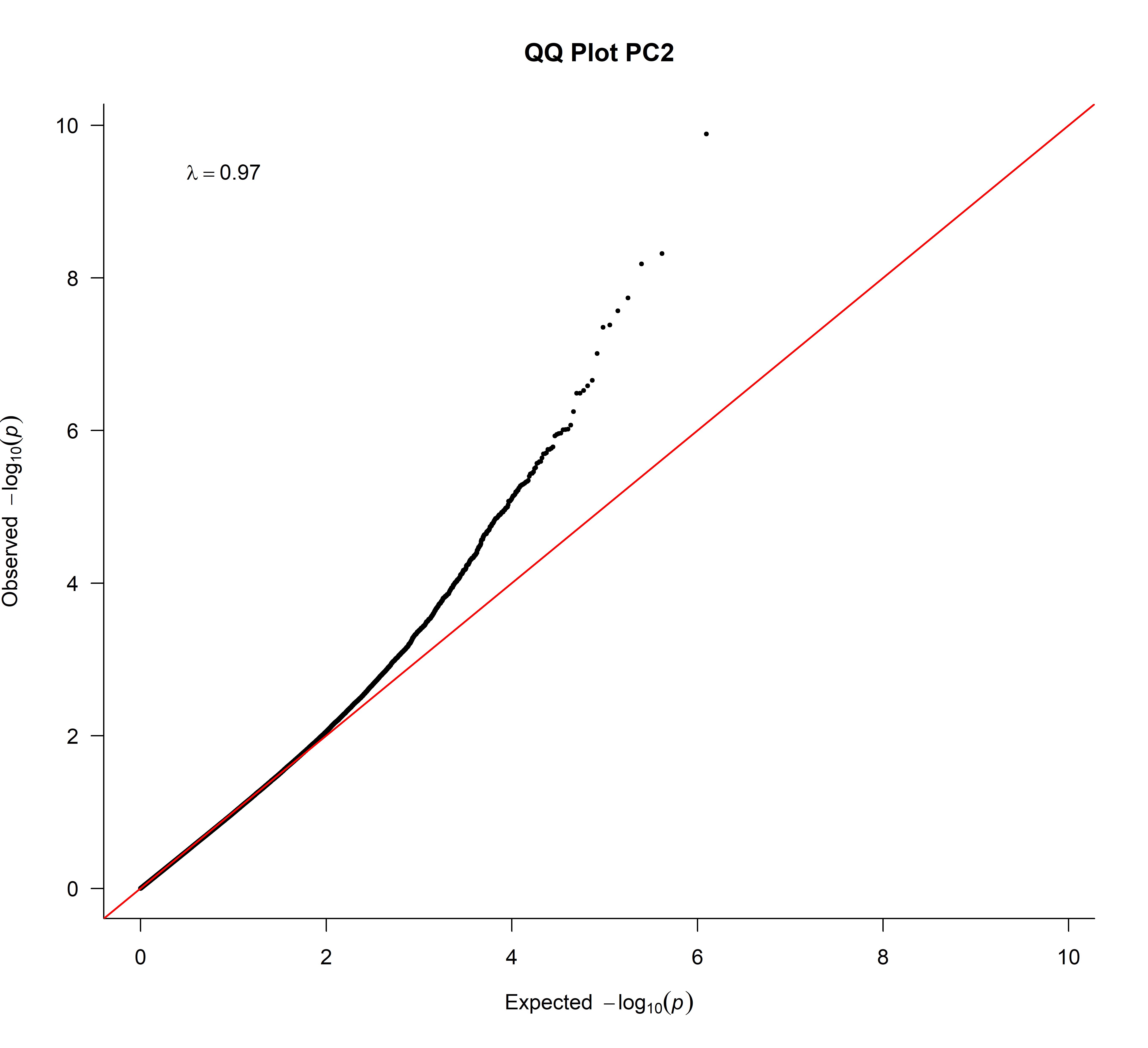

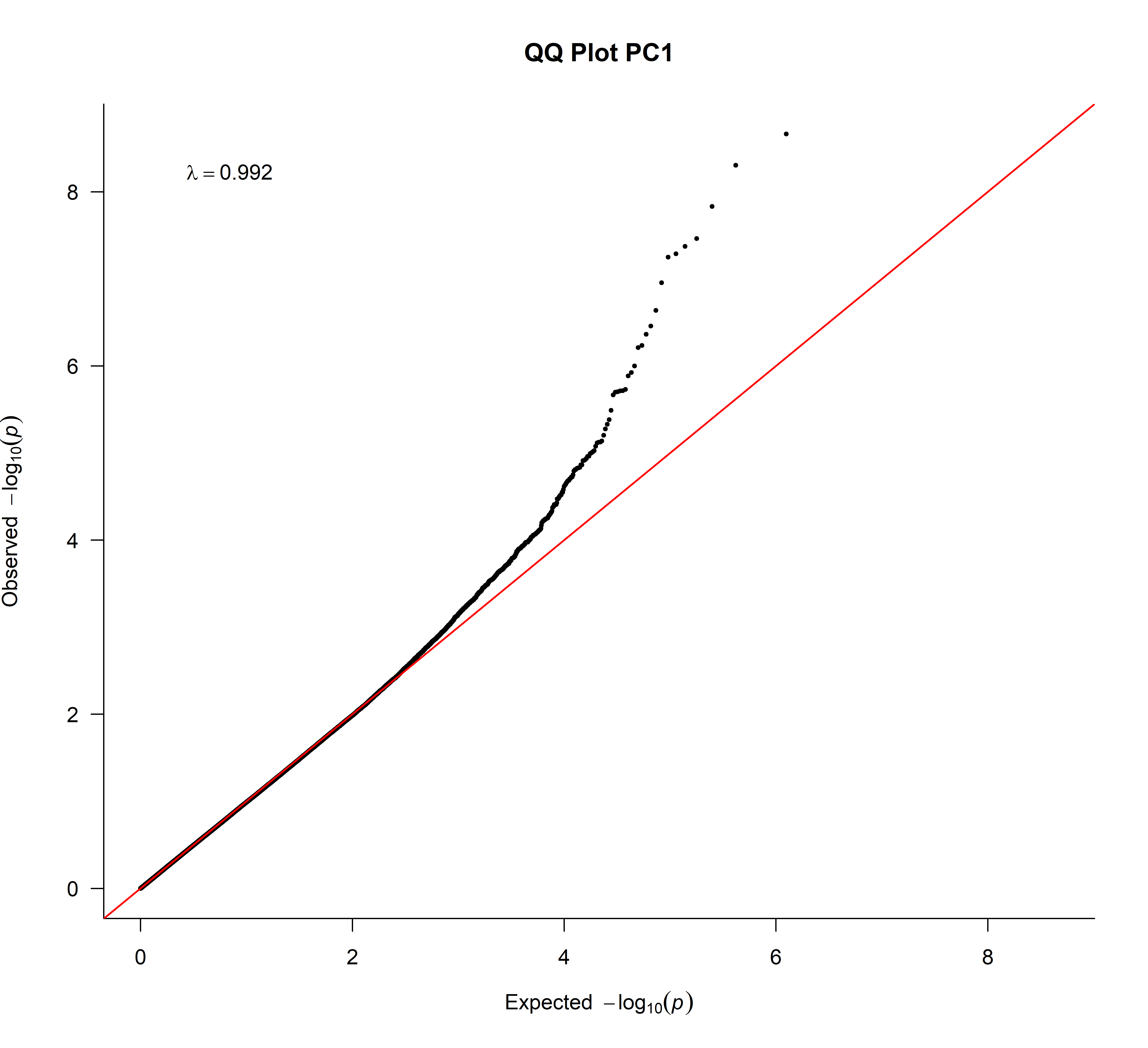

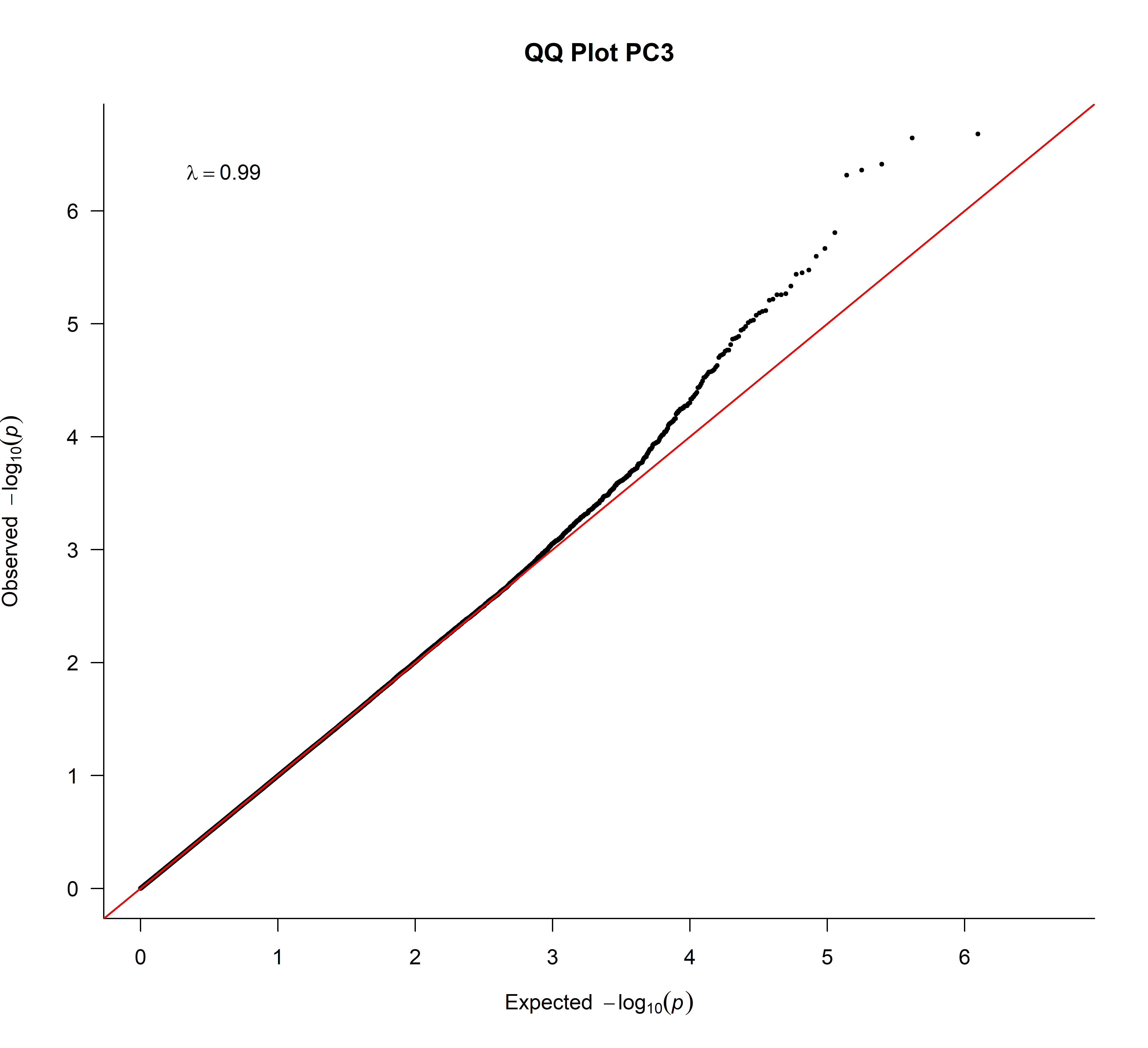

Supplement: Supplementary file 2 — Supplementary Material 2 [file 41598_2025_32828_MOESM2_ESM.docx]
