# Supplementary material for: Genome-wide association study identifies novel variants in olfactory, vitamin A, vitamin B, and cadherin pathways associated with learning and memory
Source: Sci Rep. 2025 Dec 18;16:2911. doi: 10.1038/s41598-025-32828-8 (PMC12830837; doi:10.1038/s41598-025-32828-8)
Supplement: Supplementary file 3 — Supplementary Material 3 [file 41598_2025_32828_MOESM3_ESM.docx]

**Supplementary Figure 2: Population genetic substructure compared to self-reported ethnicity.** A: Scatterplot of PC1 v PC2 of the genetic substructure with minimal clustering of self-reported ethnicity evident. B: Scatterplot of PC1 v PC3 of the genetic substructure. C: Scatterplot of PC2 v PC3 of the genetic substructure. D: Scree plot of eigenvalues depicting variance of explained by PCs of genetic substructure, inflection point denoted by red dashed line.


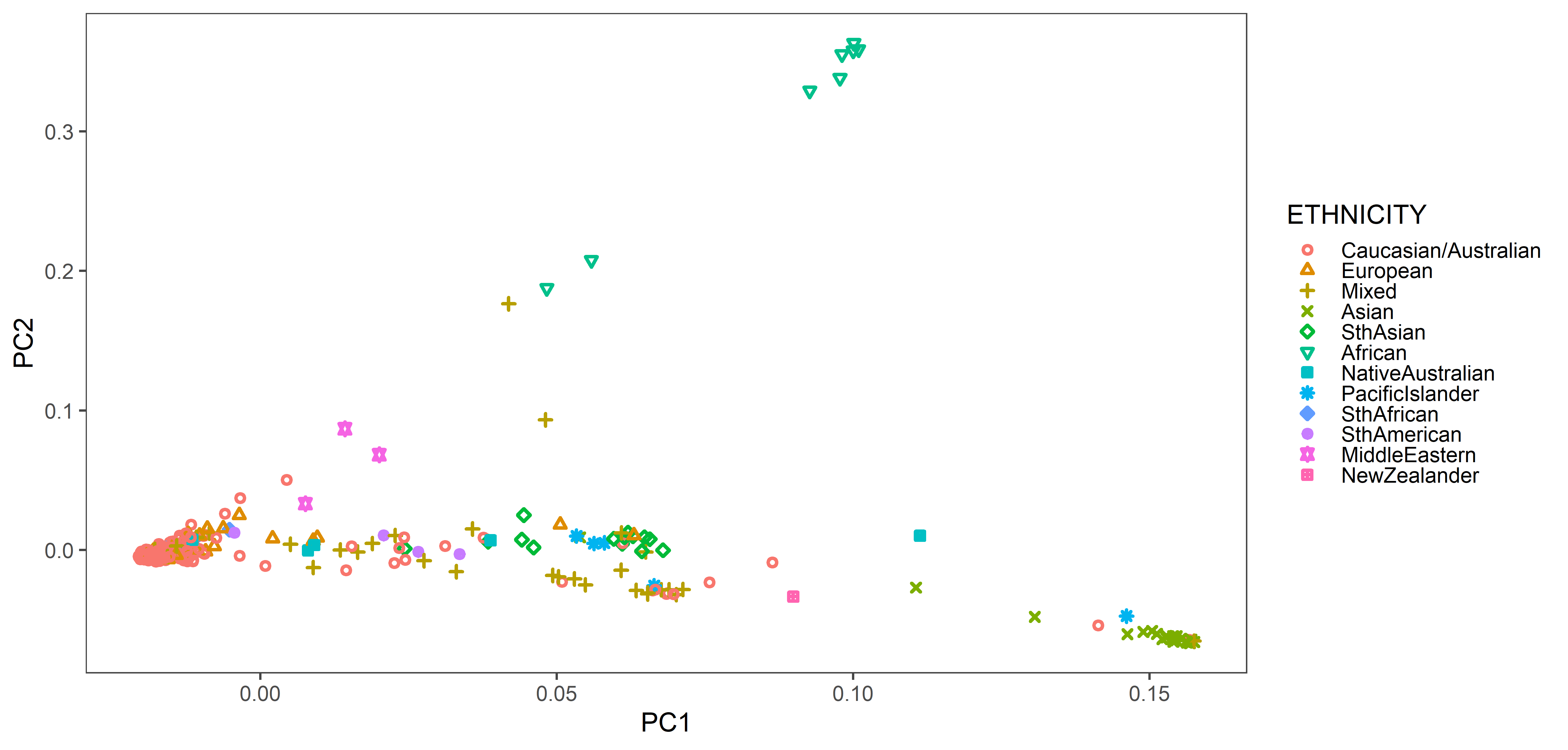

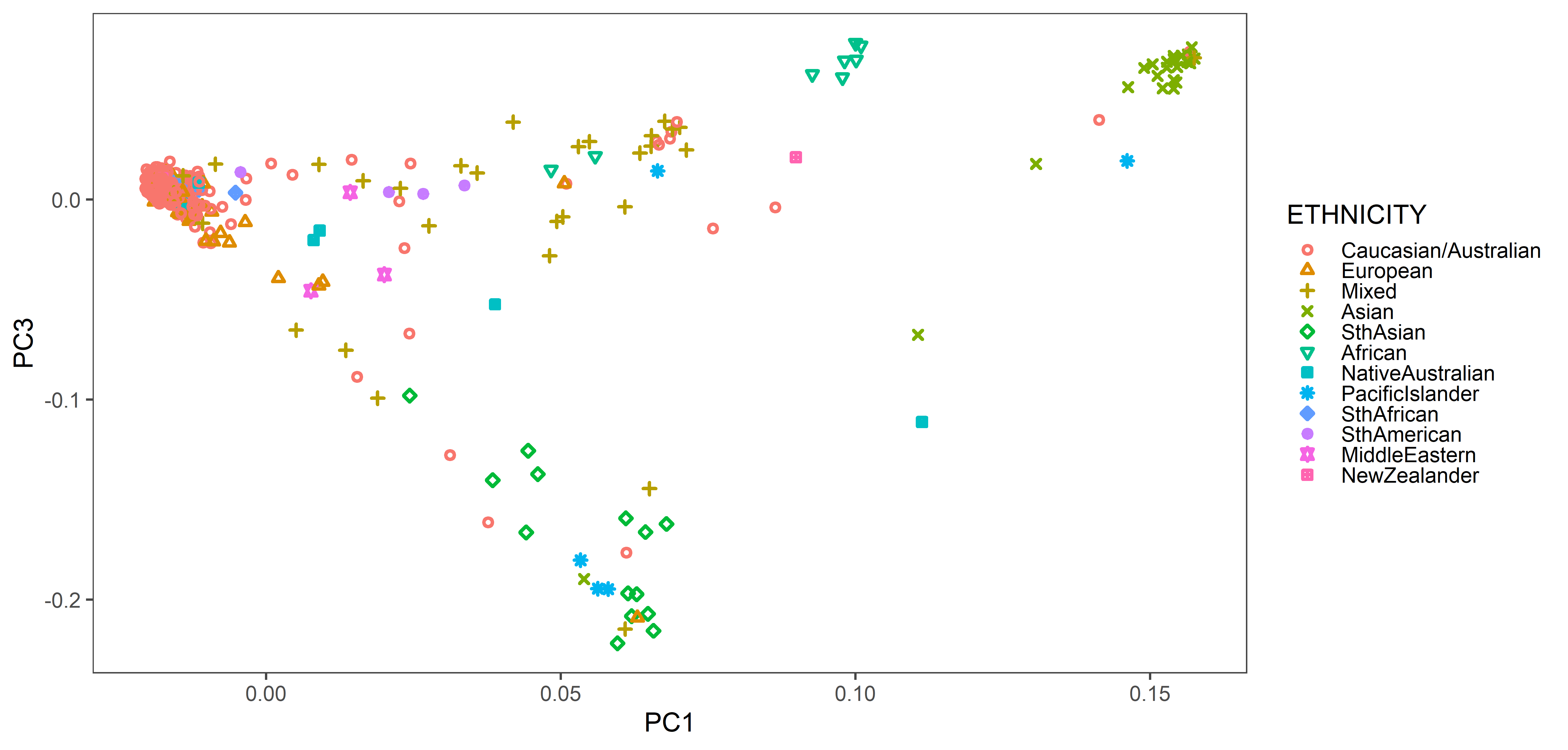

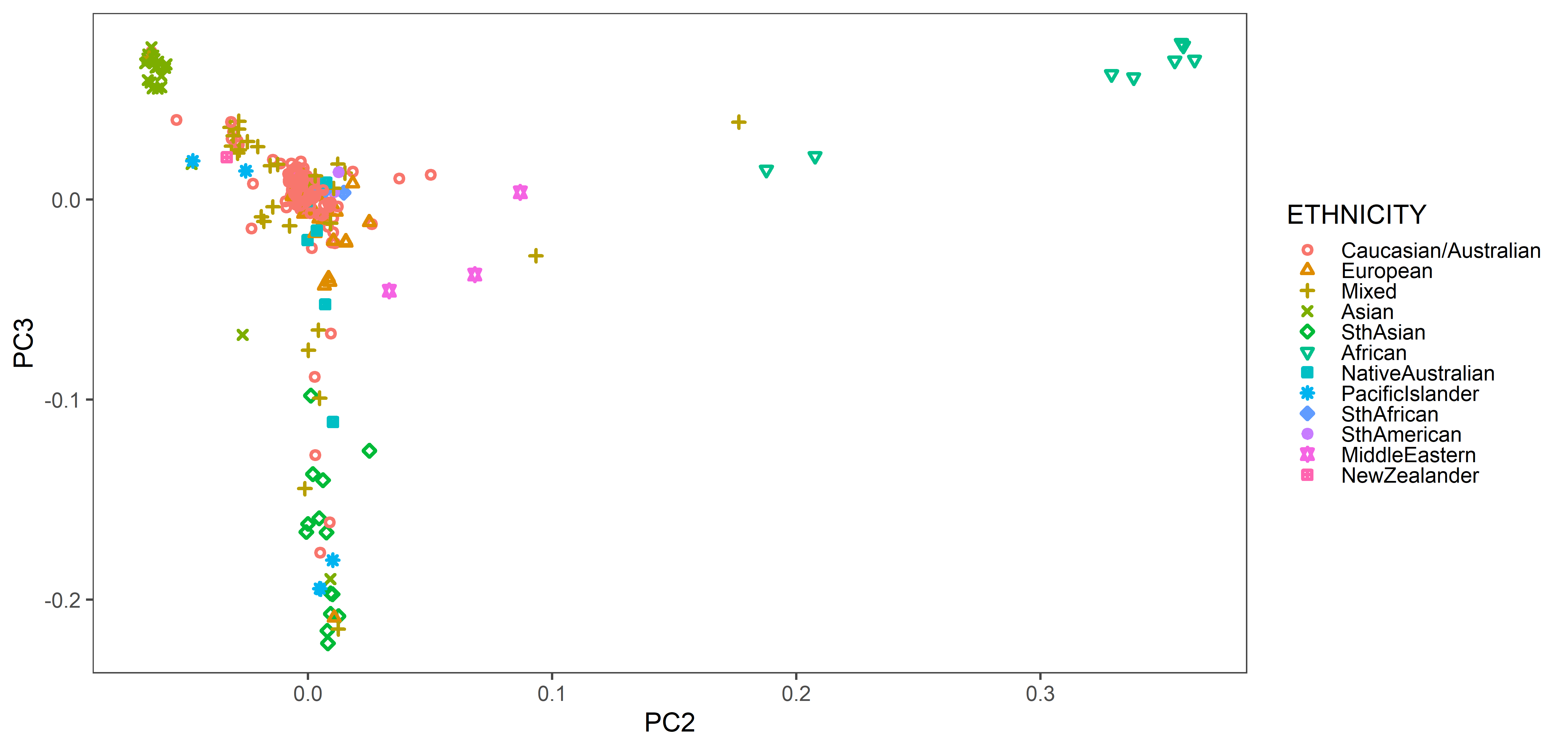

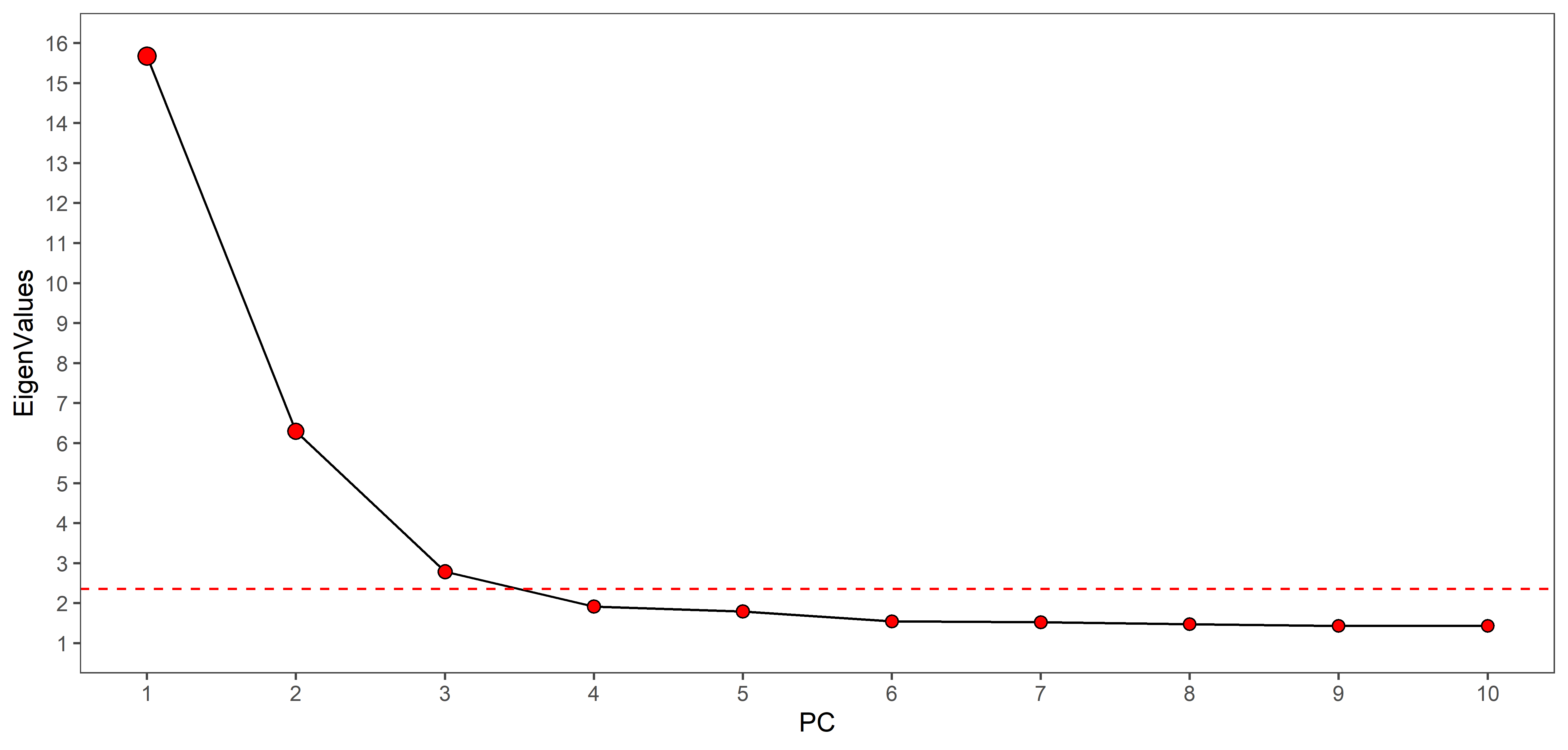


**D**

**C**

**B**

**A**
