# Supplementary material for: Genome-wide association study identifies novel variants in olfactory, vitamin A, vitamin B, and cadherin pathways associated with learning and memory
Source: Sci Rep. 2025 Dec 18;16:2911. doi: 10.1038/s41598-025-32828-8 (PMC12830837; doi:10.1038/s41598-025-32828-8)

**Supplementary Figure 4:** Manhattan plots for PC3 phenotype. The genome wide significance threshold (p< 5 × 10^−8^) is indicated by the red line. The blue line indicates the suggestive threshold (p<1x10^-5^).


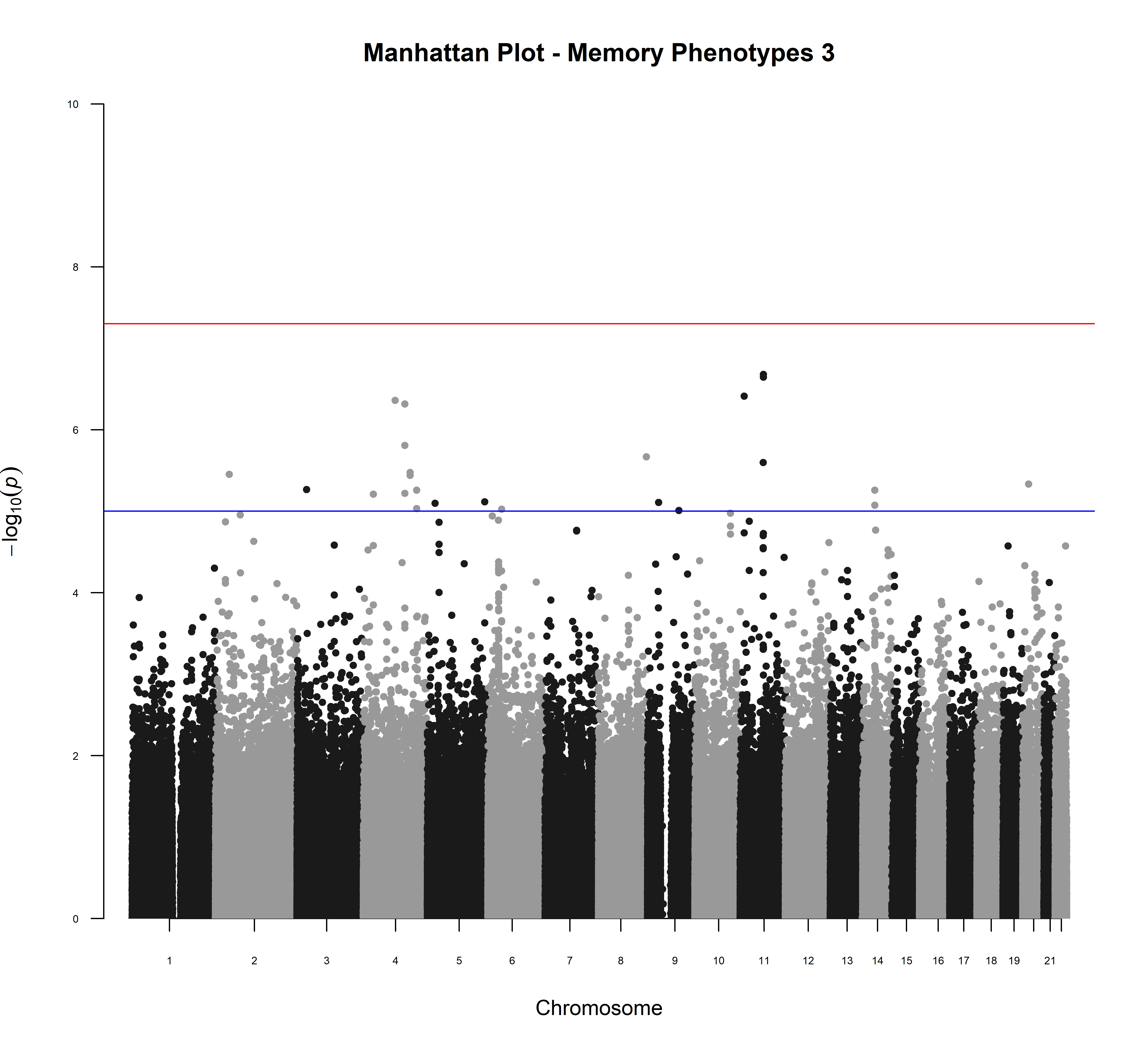

Supplement: Supplementary file 5 — Supplementary Material 5 [file 41598_2025_32828_MOESM5_ESM.docx]
